# Recent advances of traditional Chinese medicine against cardiovascular disease: overview and potential mechanisms

**DOI:** 10.3389/fendo.2024.1366285

**Published:** 2024-09-30

**Authors:** Junting Dai, Lulu Qiu, Yi Lu, Miao Li

**Affiliations:** ^1^ Department of Pharmacy, The Second Hospital of Dalian Medical University, Dalian, China; ^2^ Department of Cardiovascular, The Second Affiliated Hospital of Dalian Medical University, Dalian, China

**Keywords:** cardiovascular disease, traditional Chinese medicine, heart function, therapeutic mechanisms, gut microbiota

## Abstract

Cardiovascular disease (CVD) is the leading cause of human mortality worldwide. Despite Western medicine having made encouraging results in the clinical management of CVD, the morbidity, mortality, and disability rates of the disease remain high. Modern pharmacology has confirmed that traditional Chinese medicine (TCM), characterized by its multi-component, multi-target, and integrity, plays a positive and important role in the prevention and treatment of various CVDs in China, which has notable advantages in stabilizing disease, improving heart function, and enhancing the quality of life. Importantly, TCM is gradually being accepted by the international community due to its low cost, high safety, versatile bioactivity, and low toxicity. Unfortunately, comprehensive studies on the therapeutic effect of TCM on CVD and its mechanisms are very limited, which may restrict the clinical application of TCM in CVD. Therefore, this review is performed to analyze the pathogenesis of CVD, including inflammatory response, oxidative stress, mitochondrial dysfunction, pyroptosis, ferroptosis, dysbiosis of gut microbiota, *etc.* Moreover, we summarized the latest progress of TCM (formulas, extracts, and compounds) in curing CVD according to published literature from 2018 to 2023, as well as its mechanisms and clinical evidence. In conclusion, this review is expected to provide useful information and reference for the clinical application of TCM in the prevention and treatment of CVD and further drug development of CVD.

## 1 Introduction

Cardiovascular disease (CVD) is the diseases of the circulatory system, including disorders of the heart and blood vessels. As a chronic progressive condition, CVD is characterized by high morbidity, mortality, hospitalization, and disability rates, causing a huge economic and health burden worldwide (1, 2). According to the World Health Organization, CVD was the leading cause of the highest number of deaths in 2019 (3), and about 23 million CVD-related deaths in 2030 (4). Meanwhile, CVD remains the predominant cause of human mortality in China (5) and Western countries (6). Recent studies have confirmed that the occurrence and progression of CVD are the results of the interaction of genetic and environmental factors, and common risk factors include age, obesity, tobacco use, alcohol consumption, dyslipidemia, hypertension, diabetes (7–12), *etc.* Meanwhile, other studies have found that air pollution and circadian syndrome as contributing factors to CVD (13, 14). In addition, numerous studies have demonstrated that oxidative stress, inflammatory response, programmed cell death (such as apoptosis and autophagy, pyroptosis, and ferroptosis), and intestinal flora disorders were associated with the abnormalities of structural and functional in the cardiovascular system (15–17). Currently, surgery and drugs are commonly used in the clinical management of various CVDs, but surgical procedures are both risky and expensive. Besides, the effectiveness of cardiovascular drugs decreases with prolonged use and is accompanied by adverse side effects, which has become a major problem that needs to be urgently addressed in the Western medical treatment of CVD. Therefore, the pathogenesis of CVD needs to be further explored and effective prevention and treatment strategies need to be developed.

Traditional Chinese medicine (TCM) is an accumulation of the Chinese Nation’s clinical experience for thousands of years, characterized by comprehensive resources and low cost, and has been widely used for treating various diseases in clinical practice (18, 19). TCM was an important source of modern drug development for more than 2,000 years. More interestingly, TCM has become increasingly popular in many developed countries (20), such as Australia and the United States, because of its unique advantages including low adverse effects, stable efficacy, and a wide range of targets. Modern medical studies have demonstrated that TCM (including formulas, extracts, and compounds) possessed significant effects on the treatment of CVD, and TCM treatments are well tolerated by patients with CVD (21). Currently, the “compound Dan-Shen dropping pill”, which consists of three TCMs for the treatment of coronary heart disease and angina pectoris, was the first TCM formula in the world to complete a phase III randomized, double-blind, and international multicenter clinical trial approved by the U.S. Food and Drug Administration (NCT00797953) and this drug was widely used in Australia after being approved by the Australian Therapeutic Goods Administration. Meanwhile, the standard of Panax notoginseng extracts has been incorporated into the German Drug Code for the benefit of patients with CVD. Functionally, TCM can exert cardioprotective effects through multiple targets on oxidative stress, inflammation, autophagy, lipid metabolism, cardiomyocyte/vascular endothelial cell function, and gut microbiota (22–24), which compensates for the lack of a single drug model for the treatment of CVD in clinical. Several studies have confirmed that TCM combined with Western drugs can more effectively alleviate clinical symptoms and disease progression in patients with CVD (25, 26). Importantly, with the development of omics technologies such as transcriptome, proteome, metabolome, and bioinformatics, the detailed mechanisms of TCM in the prevention and treatment of CVD have been systematically and comprehensively expanded to multiple levels such as RNA, protein, and metabolites, and also extend to the single-cell microscopic level from the perspective of time and space (27). This suggests that TCM provides new perspectives and strategies to combat various CVDs in modern society.

Currently, there are few reviews on TCM for the prevention and treatment of various CVDs. In this review, the current pathogenesis of CVD was comprehensively overviewed. Moreover, the current research on TCM (including TCM formulas, extracts, and compounds) protection against CVD was summarized and discussed based on the published literature from 2018-2023 through global and local databases including PubMed, Web of Science, and China National Knowledge Infrastructure, as well as its mechanisms and clinical efficacy, which may provide a reference for the clinical application of TCM in the treatment of CVD and a theoretical basis for the development of new drugs to combat CVD.

## 2 The pathogenesis of CVDs

The development and progression of CVD were associated with genetic mutations, obesity, environmental factors, and poor lifestyle (28, 29). Increasing evidence has demonstrated that the possible pathogenesis of CVD includes inflammation, oxidative stress, mitochondrial dysfunction, cell death (e.g., apoptosis, ferroptosis, and pyroptosis), and gut microbiota imbalance, which would lead to cardiomyocyte injury, inflammatory response, and vascular lesions (15, 30, 31), etc.

### 2.1 Inflammation

**Figure 1:**
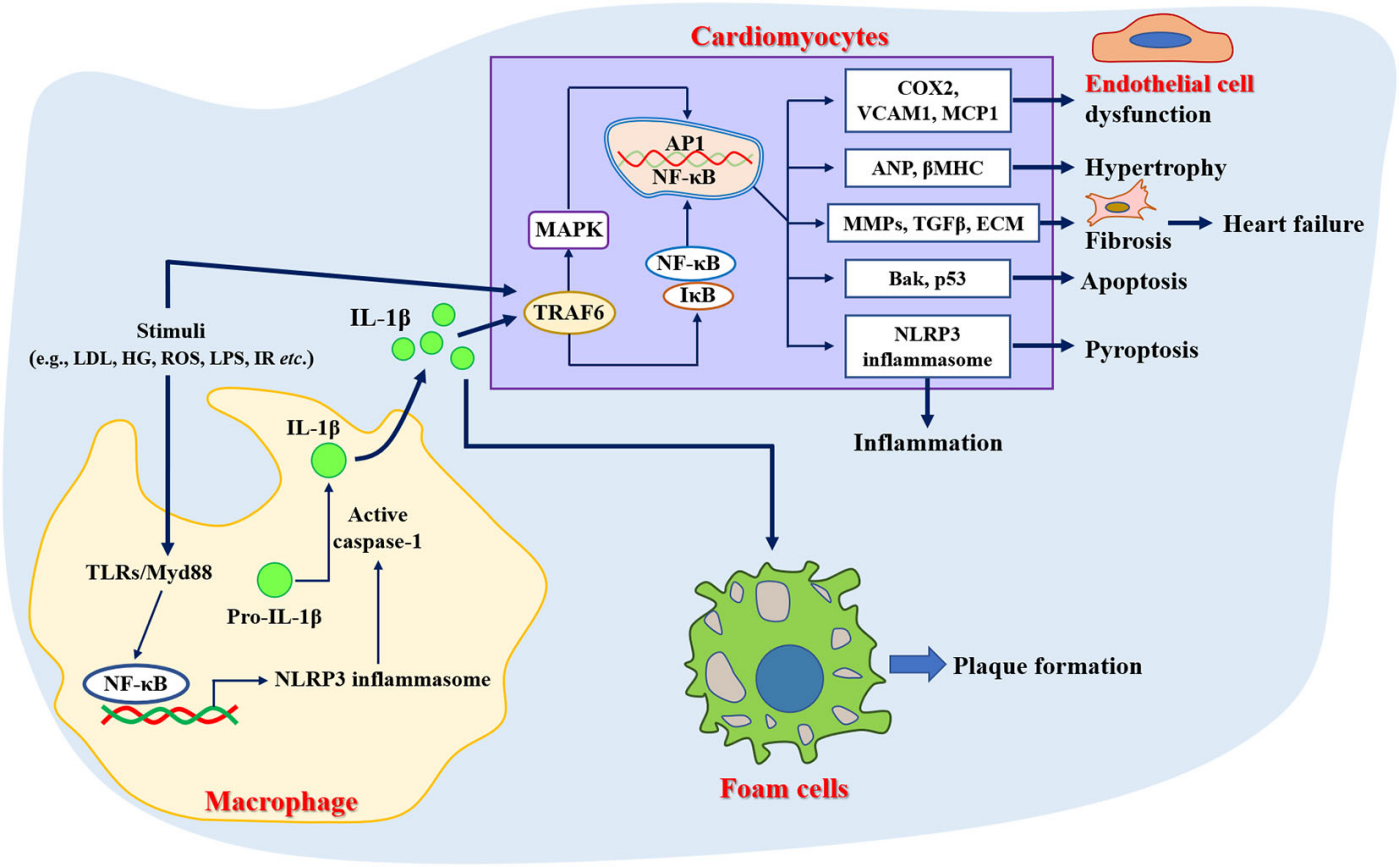
Role of inflammation in the pathogenesis of cardiovascular diseases. ANP, Atrial natriuretic peptide; Bak, Bcl-2 antagonist/killer; COX2, Cyclooxygenase 2; ECM, Extracellular matrix; HG, High glucose; LDL, Low-density lipoprotein; LPS, Lipopolysaccharide; MCP1, Monocyte chemotactic protein 1; NLRP3, Nucleotide-binding oligomerization domain-like receptor family pyrin domain-containing 3; ROS, Reactive oxygen species; TGFβ, Transforming growth factor beta; TLRs, Toll-like receptors; TRAF6, Tumor necrosis factor receptor-associated factor 6; VCAM1, Vascular cell adhesion molecule 1; βMHC, Beta-myosin heavy chain.

Inflammation plays an important role in the pathogenesis of various CVDs (32), and anti-inflammatory therapies have proven beneficial in several recent clinical trials (33, 34). Increased incidence of cardiovascular events has also been shown in patients with chronic inflammatory diseases such as rheumatoid arthritis, systemic lupus erythematosus, psoriasis, inflammatory myopathies, and inflammatory bowel disease (35). Evidence suggested that the upregulation of circulating C reactive protein resulted in a greater risk of incident acute myocardial infarction (36) or cerebrovascular events (37). Previous studies have shown that atherosclerosis is a low-grade and aseptic inflammatory disease (38). For example, Mai et al. (39) demonstrated that nucleotide-binding oligomerization domain-like receptor family pyrin domain-containing 3 (NLRP3) inflammasome was a key driver of atherosclerosis. Meanwhile, the inflammatory response was considered to be a trigger for the developmental process of atrial fibrillation (40). Over-activation of NLRP3 inflammasome was directly associated with hospitalization rates in patients with cardiac insufficiency and dilated cardiomyopathy, accompanied by cellular scorching of cardiomyocytes (41). In addition, it has also been demonstrated that inhibition of the inflammatory response or NLRP3 gene deletion improved cardiac remodeling and reduced proinflammatory cytokines secretion and fibrotic processes (42, 43), as well as attenuated angiotensin II (Ang II)-induced hypertension (44). Taken together, inflammation was involved in the pathogenesis of several CVDs (**Figure 1**), which also provides new strategies for the prevention and management of CVD.

### 2.2 Oxidative stress

**Figure 2:**
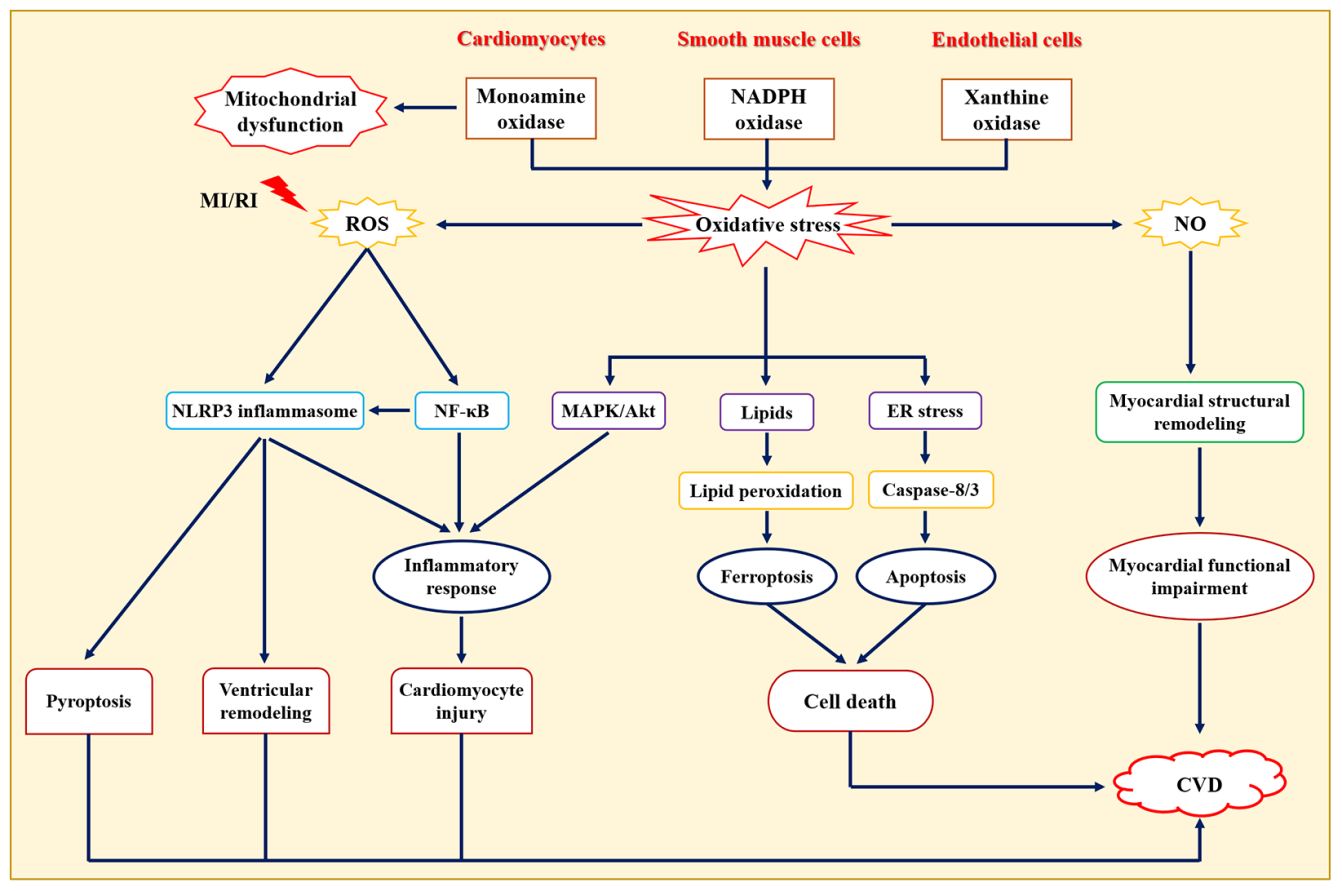
Role of oxidative stress in the pathogenesis of cardiovascular diseases. NO: one of the members of reactive nitrogen, damages cardiomyocytes through direct cytotoxicity or generates ONOO^−^ with O^2−^ to cause cardiomyocyte damage. CVD, Cardiovascular diseases; ER, Endoplasmic reticulum; MAPK, Mitogen-activated protein kinase; MI/RI, Myocardial ischemia/reperfusion injury; NF-κB, Nuclear transcription factor-κB; NLRP3, Nucleotide-binding oligomerization domain-like receptor protein 3.

Oxidative stress is a pathological state of reactive oxygen species (ROS) accumulation caused by excessive production of oxygen free radicals or impaired intracellular antioxidant defense systems (45). Normal physiological state of ROS levels contributes to the maintenance of cardiovascular homeostasis (46), while excessive and/or sustained increases in ROS production play an important role in the pathological statute of CVD (**Figure 2**), such as atherosclerosis, hypertension, myocardial ischemia-reperfusion injury, arrhythmia, heart failure, and acute myocardial infarction (47). Of note, oxidative stress has emerged as a new target for the prevention and treatment of CVD (48). It has also been found that common CVD risk factors contribute to a sustained increase in ROS production in the vascular wall (49). Functionally, oxidative stress not only promotes lipid peroxidation, protein and enzyme denaturation, DNA damage, and severe functional impairment of vascular endothelial cells and cardiomyocytes, but also participates in the pathogenesis of hypertension, myocardial ischemia-reperfusion injury, atherosclerosis, and other CVDs by regulating inflammation and stimulating vascular smooth muscle cell proliferation (50). In addition, endogenous antioxidant enzymes (e.g., superoxide dismutase, glutathione peroxidase, catalase, glutathione S-transferase, and peroxidase) and exogenous antioxidants may act by scavenging free radicals and exerting anti-CVD activities. For example, overexpression of glutathione peroxidase 4 (GPX4) inhibited atherosclerosis progression in apolipoprotein E-deficient (ApoE^-/-^) mice (51). Giam et al. (52) showed that the antioxidant NAC attenuated cardiac injury and prevented cardiac fibrosis which improved cardiac function in mice with heart failure.

### 2.3 Mitochondrial dysfunction

**Figure 3:**
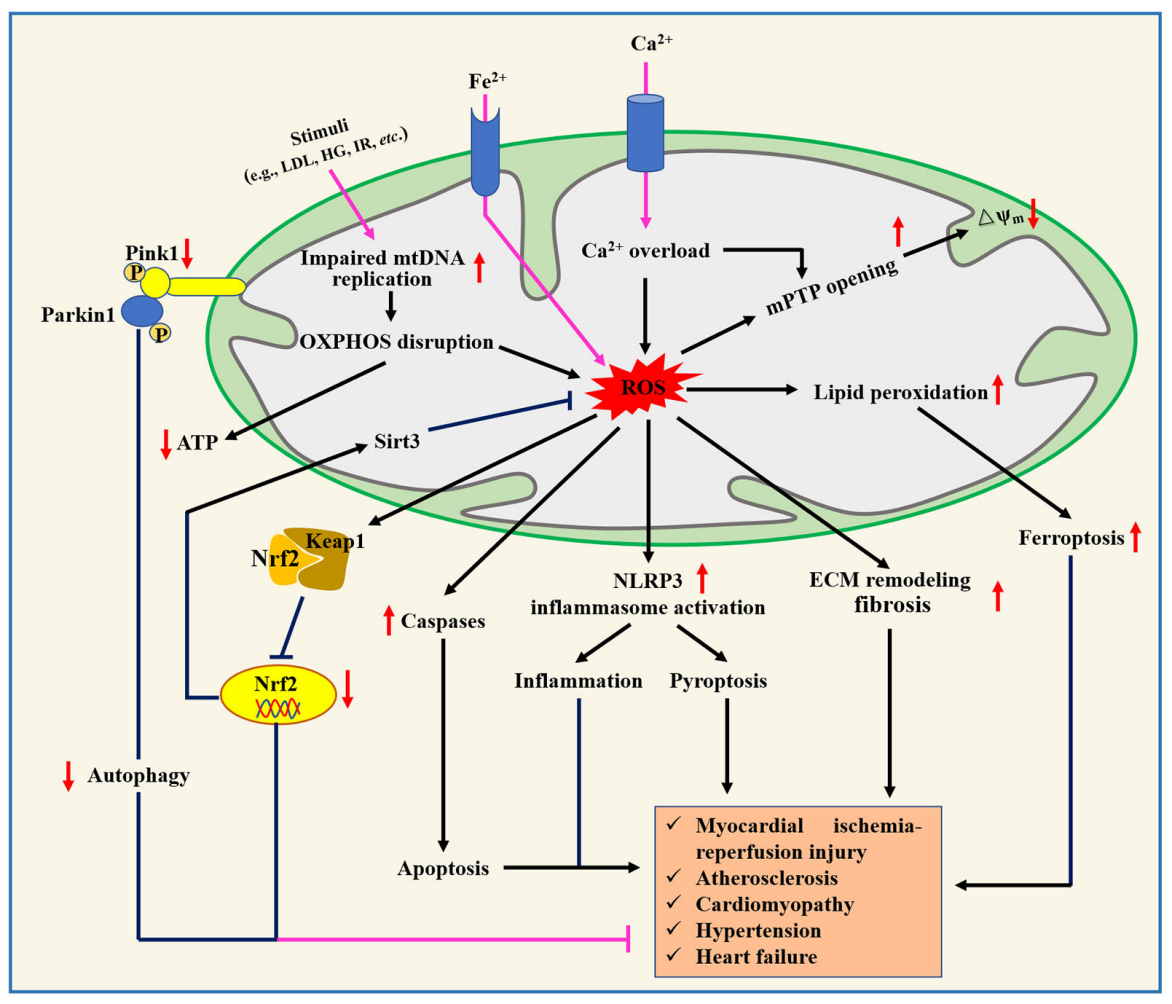
Role of mitochondrial dysfunction in the pathogenesis of cardiovascular diseases. ECM, Extracellular matrix; HG, High glucose; IR, ischemia/reperfusion; Keap1, Kelch-like ECH-associated protein 1; LDL, Low-density lipoprotein; mPTP, Mitochondrial permeability transition pore; Nrf2, Nuclear factor erythroid 2-related factor 2.

Mitochondria, a key site of cellular metabolism for ATP production, provides enough energy for the contraction and diastole of human cardiomyocytes, but mitochondrial dysfunction accelerates the occurrence and progression of CVD (**Figure 3**). For example, mitochondrial dysfunction in macrophages contributes to inducing inflammation and inhibiting repair after myocardial infarction, but mitochondrial-targeted ROS scavenging alleviates these phenomena and reduces death after myocardial infarction in mice (53). Currently, mitochondrial dysfunction, mitochondrial DNA and nuclear DNA gene mutation, and the presence of mutant proteins associated with mitochondria are considered to be non-negligible causes of CVD pathogenesis (54). For instance, four mitochondrial DNA mutation genes (e.g., MT-RNR1, MT-TL1, MT-TL2, and MT-CYB) have been reported to be connected with atherosclerosis progression (55). Functionally, mutations in the mitochondrial genome and nuclear genome may disrupt mitochondrial homeostasis, leading to excessive ROS production and reducing oxidative phosphorylation capacity, which are risk factors for CVD (56). For example, specific targeted antioxidant treatments that reduced ROS production and enhanced ROS scavenging have been shown to alleviate impaired mitochondrial-induced oxidative stress (57). Jacinto et al. (58) showed that the overproduction of mitochondrial ROS promoted atherosclerosis progression by triggering DNA fragmentation and cell apoptosis. Moreover, mitophagy plays an important regulatory role in maintaining cellular homeostasis, whereas mitophagy damage predisposes to cause abnormal function of cardiovascular-derived cells (59). Notably, several intervention strategies ameliorate CVD by improving four important characteristics of mitochondria, such as scavenging mitochondrial ROS (60), mitochondrial DNA editing or mitochondrial replacement therapy (61), increased oxidative phosphorylation (62), and enhanced mitophagy (63). Therefore, maintaining normal mitochondrial function has the potential to be used as an effective therapeutic strategy for CVDs.

### 2.4 Pyroptosis

**Figure 4:**
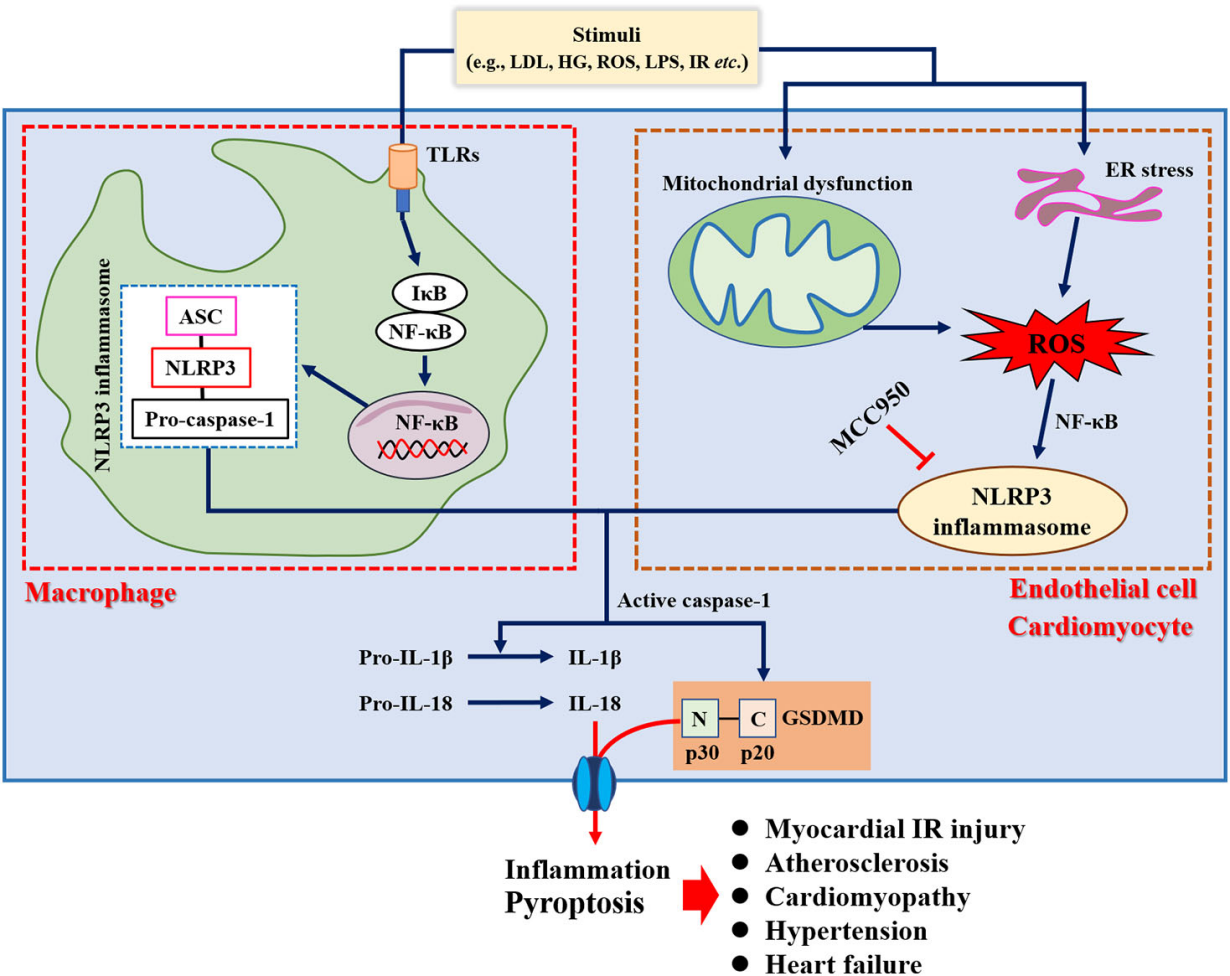
Role of pyroptosis in the pathogenesis of cardiovascular diseases.

Pyroptosis, a form of programmed cell death, is closely related to the inflammatory response, mediated by the Gasdermin protein, and dependent on caspase activity (64). Pyroptosis is typically characterized by the swelling and rupture of cell membranes, the release of pro-inflammatory factors, and cell contents from the plasma membrane to the extracellular environment (65), which aggravates inflammatory response. Recent studies have shown that pyroptosis was involved in the development and progression of several CVDs (**Figure 4**), including atherosclerosis, diabetic cardiomyopathy, myocardial infarction, myocardial ischemia-reperfusion injury, myocarditis (66), etc. Mechanistically, NLRP3 inflammasome activated caspase-1 and triggered an inflammatory cascade, which plays an important role in pyroptosis (67). For example, NLRP3 inhibitor MCC950 has the potential to prevent NLRP3-related diseases, such as cardiac hypertrophy (68), hypertension (69), atherosclerosis (70), and myocardial injury (71). Jin et al. (72) showed that caspase-1 inhibitor VX765 ameliorated mitochondrial damage induced by the NLRP3 inflammasome activation and inhibition of vascular inflammation in both low-density lipoprotein receptor-deficient (Ldlr^-/-^) and ApoE^-/-^ mice. These results suggested that inhibition of pyroptosis may provide a new avenue for the treatment and management of CVDs.

### 2.5 Ferroptosis

**Figure 5:**
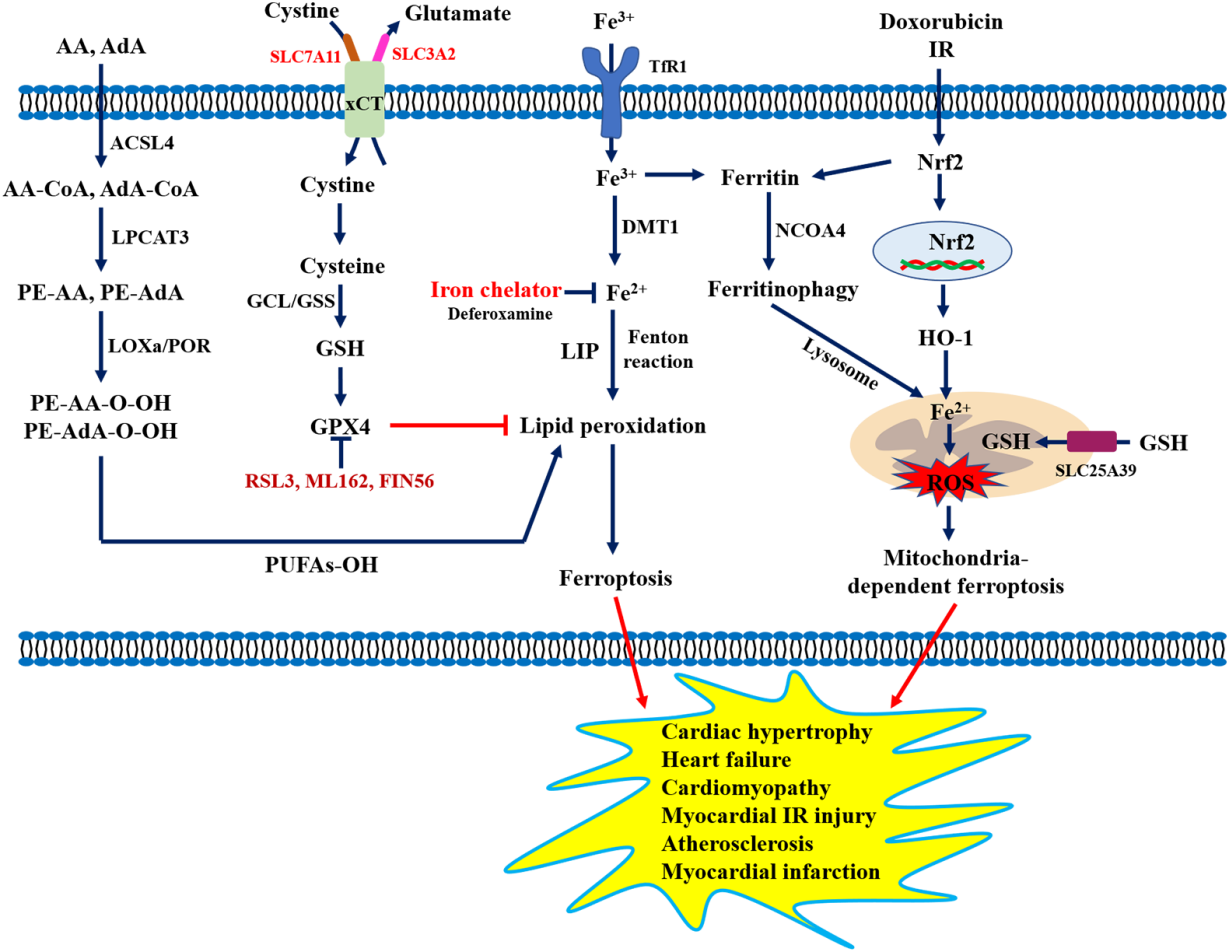
Role of ferroptosis in the pathogenesis of cardiovascular diseases. AA, Arachidonic acid; ACSL4, Long-chain fatty acyl-CoA synthase 4; AdA, Adrenal acid; DMT1, Divalent metal transporter 1; FfR1, Transferrin receptor 1; GCL, Glutamate-cysteine ligase; GPX4, Glutathione peroxidase 4; GSH, Glutathione; GSS, Glutathione synthase; HO-1, Heme oxygenase 1; LPCAT3, Lysolecithin acyltransferase 3; LOXs, Lipoxygenases; NCOA4, Nuclear receptor coactivator 4; POR, Cytochrome P450 oxidoreductase; PUFAs, Polyunsaturated fatty acids; SLC7A11, Solute carrier family 7 member 11; xCT, System X^c-^.

Ferroptosis is a new type of cellular iron-dependent programmed cell death, and the process mainly involves the accumulation of lipid peroxidation products and lethal ROS (73). Increasing evidence has demonstrated that ferroptosis was morphologically, biochemically, and genetically distinct from cell apoptosis, necrosis, and autophagy (74), which was mainly characterized by impaired cell membrane integrity, mitochondrial atrophy, normal nuclei, and a significant decrease in the levels of GPX4, glutamate-cystine antiporter system components (SLC3A2 and SLC7A11), and coenzyme II. Available studies have shown that ferroptosis was closely associated with the development of various CVDs including cardiomyopathy, myocardial ischemia-reperfusion injury, heart failure, myocardial infarction, vascular injury, and atherosclerosis (75). For example, Wang et al. (76) reported that increased levels of lipid peroxidation and reduced SLC7A11 levels were observed in the development of diabetic cardiomyopathy. Bai et al. (77) found that ferrostatin-1 (Fer-1, ferroptosis inhibitor) alleviated atherosclerotic lesions by reducing iron accumulation and lipid peroxidation, and enhancing the expression of GPX4 and SLC7A11 in a high-fat diet (HFD)-fed ApoE^-/-^ mice. Another study showed that the inactivation of the Nrf2/GPX4 pathway could aggravate doxorubicin-induced cardiomyopathy by promoting cardiomyocyte ferroptosis (78). Importantly, three types of iron chelators (e.g., deferiprone, deferoxamine, deferasirox) have been used in clinical practice for the treatment of iron overload cardiomyopathy (79). Although many preclinical studies suggest that pharmacological regulation of ferroptosis and genetic inhibition of iron uptake are promising treatment strategies for CVD (**Figure 5**), the underlying mechanism and regulatory networks need to be fully investigated during the pathological process of CVD, which will provide new ideas and strategies for the prevention and treatment of CVD.

### 2.6 Gut microbiota and metabolomics

**Figure 6:**
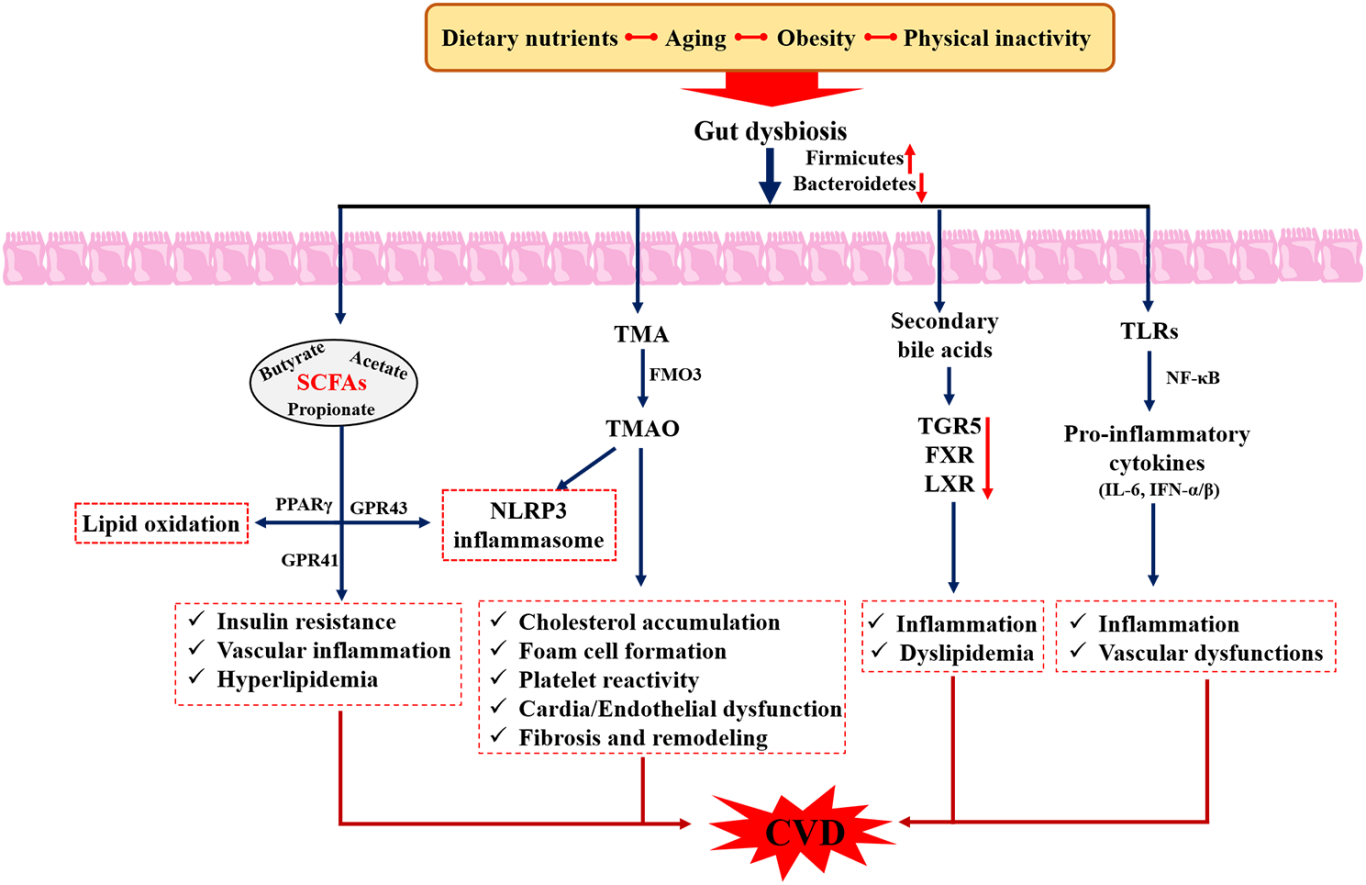
Role of gut microbiota in the pathogenesis of cardiovascular diseases. SCFAs, Short chain fatty acids; LPS, Lipopolysaccharides; TGR5, Takeda G-protein-coupled receptor 5; FXR, farnesoid X receptor; TMAO, trimethylamine-N-oxide; TMA, trimethylamine.

Gut microbiota refers to the large number of commensal microorganisms living in the human intestinal tract, which mainly consists of *Firmicutes*, *Bacteroidetes*, *Proteobacteria*, *Fusobacteria*, and *Actinobacteria* at the phylum level, but its balance is easily disturbed by food intake, lifestyle, and environment (80). Functionally, the gut microbiota can form the intestinal epithelial barrier, regulate intestinal immunity, and prevent the invasion of pathogenic bacteria and metabolic abnormalities (81), which are essential for human health. Numerous studies have demonstrated that dysbiosis of intestinal bacteria and its metabolites, such as Trimethylamine oxide (TMAO), lipopolysaccharides (LPS), short-chain fatty acids (SCFAs), and bile acids, were closely associated with the development of CVD (82), and targeting the gut microbiota was expected to be a potential new target for the treatment of CVD (**Figure 6**). For example, Jie et al. (83) reported that patients with atherosclerotic cardiovascular disease (ACVD) possessed an increased relative abundance of *Enterobacteriaceae* and *Streptococcus* spp., which contributed to aggravating ACVD as well as other diseases. In another survey, high levels of *Prevotella*, *Hungatella*, and *Succinclasticum* and low levels of *Lachnospiraceae* family and *Faecalibacterium* were observed in patients with heart failure (84). Meanwhile, elevated plasma levels of TMAO were positively associated with stroke (85), hypertension (86), and atherosclerosis (87), as well as increased cardiovascular events (88), suggesting that reducing intake of dietary TMAO precursors was an effective strategy to decrease the risk of CVD. The above studies suggest that gut microbiota serves as a “microbial organ” that affects cardiovascular health and the “gut-heart” axis is a potential avenue in the prevention and treatment of CVD.

### 2.7 Others

Except for the pathogenesis mentioned above, researchers believe that CVD is associated with endoplasmic reticulum stress (ERS) (89), autophagy deficiency (90), diabetes (91), metabolic syndrome (92), etc. Moreover, searching for biomarkers used to determine the occurrence and progression of CVDs and revealing their mechanisms are of great clinical significance for the early diagnosis and treatment of CVD. Meanwhile, the exploration of assessment tools for the early identification of people at high risk of CVD is an important guarantee to reduce cardiovascular mortality. However, the drugs developed to address this pathogenesis can only alleviate the symptoms of CVD, but cannot inhibit or reverse CVD progression. Therefore, elucidating the pathogenesis of CVD remains a key clinical problem that needs to be addressed. Of note, understanding the pathogenesis of CVD may provide effective biomarkers and pathways for subsequent therapeutic and new drug development.

## 3 TCM in the treatment of CVD

**Figure 7:**
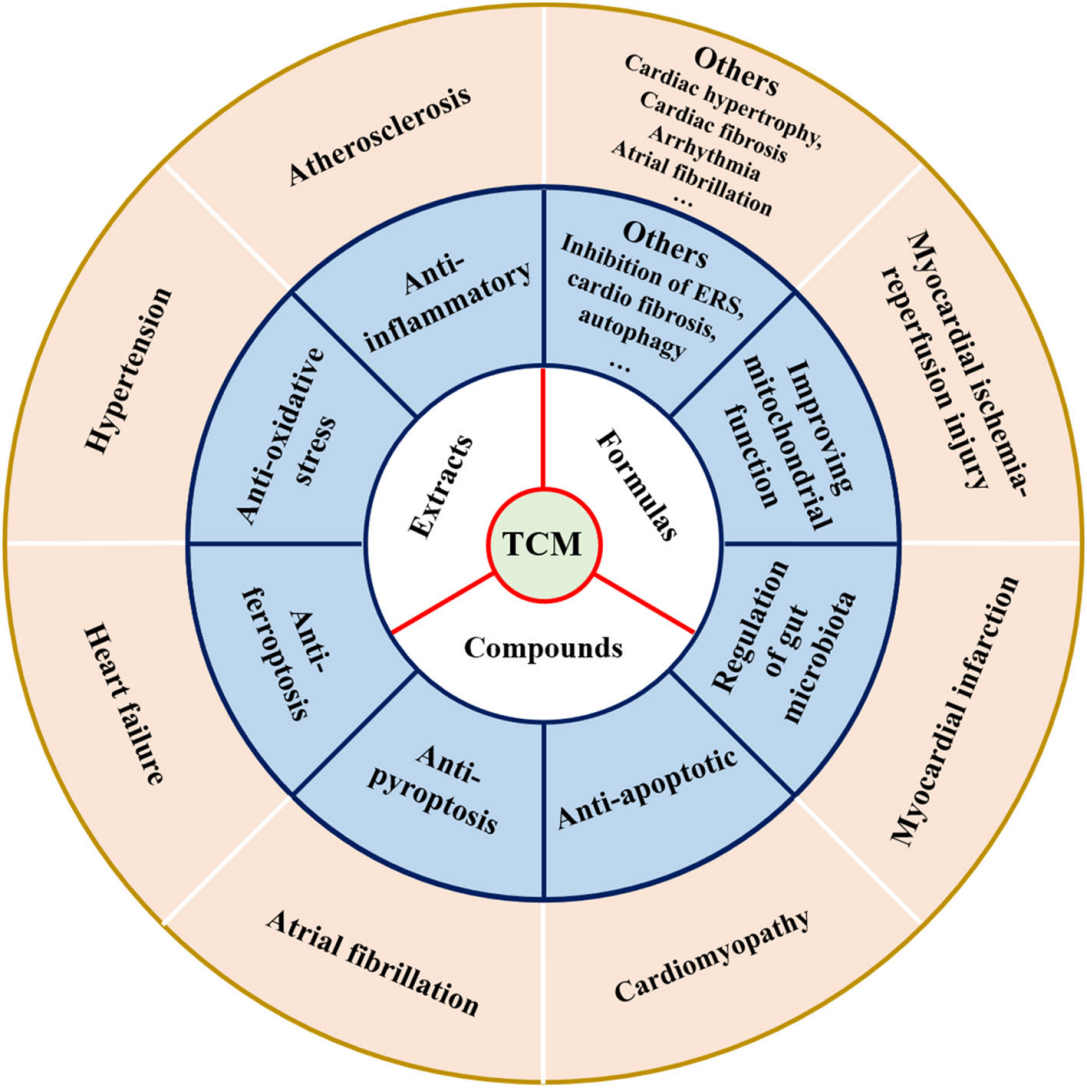
Therapeutic effects of TCM on cardiovascular diseases and its mechanism.

With in-depth research on the pathogenesis of CVD, TCM has shown unique therapeutic advantages in CVD by virtue of its multi-component, multi-target, and integrity (93). More and more studies have demonstrated that TCM (including formulas, extracts, and compounds) exhibited a protective effect on cardiovascular (21), and mechanisms of action of TCM in preventing CVD are shown in **Figure 7** and **Tables 1**–**3**. Meanwhile, the majority of Chinese patients with CVD have been treated with TCM during the diagnosis and treatment process (94). Herein, we summarized the research progress of TCM in the treatment of various CVDs to provide a reference for the research on the complex mechanism of TCM in combating CVD.

**Table 1:** Summary of traditional Chinese medicine formulas in the prevention and treatment of various cardiovascular diseases from 2018-2023.

Prescription	Composition (In Chinese)	Evaluation model	Effects and action mechanism	Ref.
Atherosclerosis				
Buyang huanwu decoction	Huangqi, Chishao, Chuanxiong, Danggui, Dilong, Taoren, and Honghua in a ratio of 120:6:4:5:3:3:3	HFD-induced ApoE^-/-^ mice	Levels of TC, TG, LDL-c↓and HDL-c↑ Levels of TNF-α, IL-1β, IL-6, iNOS↓ NF-κB pathway↓	(196)
Huang-Lian-Jie-Du decoction	Huanglian, Huangqin, Huangbo, and Zhizi in a weight ratio of 3:2:2:3	HFD-induced ApoE^-/-^ mice ox-LDL-induced RAW264.7 cells	Carotid lesion plaques stability↑ Levels of IL-1β, IL-6, TNF-α↓ Foam cell formation↓and M2 polarization↑	(197)
Guanxinkang decoction	Huangqi, Yimucao, Danshen, Xiebai, Banxia, and Gualou in a weight ratio of 10:10:4:4:4:5	HFD-induced LDLR^-/-^ mice ox-LDL-induced RAW264.7 cells	Body weight and levels of TC, TG, LDL-c↓ Atherosclerotic plaques↓and α-SMA level↑ Levels of IL-1β, IL-6, TNF-α, LOX-1, MCP-1↓ MAPKs/NF-κB pathway↓	(198)
Qing-Xin-Jie-Yu granule	Huangqi, Danshen, Chuanxiong, Guanghuoxiang, and Huanglian in a ratio of 3:3:2:2:1	HFD-induced ApoE^-/-^ mice	Body weight and levels of TC, TG, and LDL-c↓ Levels of HDL-c↑and IL-1β, IL-6↓ The abundance of *Turicibacter* and *Roseburia*↑ The abundance of *Alistripes*, *Rikenella*, *Blautia*↓	(102)
Qing-Xin-Jie-Yu granule	Huangqi, Danshen, Chuanxiong, Guanghuoxiang, and Huanglian in a ratio of 3:3:2:2:1	HFD-induced ApoE^-/-^ mice	TC, TG, LDL-c levels, and ferroptosis↓ Levels of IL-6, IL-1β, TNF-α, Fe^2+^, ROS↓ Expression of GPX4/xCT in aorta tissues↑	(199)
Yiqihuoxue decoction	Chuanxiong, Chishao, and Xiyangshen in a ratio of 40:20:1	HFD-induced ApoE^-/-^ mice	Blood glucose and levels of TNF-α and IL-6↓ Aortic arch plaque area↓	(200)
Wu-Zhu-Yu decoction	Wuzhuyu, Shengjiang, Renshen, and Dazao in a ratio of 1:2:1:1	HFD-induced ApoE^-/-^ mice	Aortic lesion areas↓ Levels of TC, TG, LDL-c↓and HDL-c↑	(201)
Tongqiaohuoxue decoction	Shaoyao, Chuanxiong, Taoren, Honghua, Onion, Wuchizao, Ginger, and Yunmuxiang in a ratio of 16:16:48:48:12:8:48:20	HFD-induced ApoE^-/-^ mice ox-LDL-induced THP-1 cells ox-LDL-induced HUVECs	Lipid deposition, plaque formation, lipid uptake↓ Levels of ICAM-1, VCAM-1, and MCP-1↓	(202)
Si-Miao-Yong-An decoction	Rendong, Xuanshen, Danggui, and Gancao in a ratio of 3:3:2:1	HFD-induced ApoE^-/-^ mice	lipid accumulation↓and Autophagy↑ NF-κB pathway↓	(203)
Tao Hong decoction	Taoren, Honghua, Chuanxiong, Danggui, and Weilingxian in a ratio of 9:9:9:9:9	HFD-induced ApoE^-/-^ mice	Plaque area and Levels of inflammatory cytokines↓ PI3K/Akt/p38 pathway↓	(204)
Bunao-Fuyuan decoction	Huangqi, Baizhi, Chishao, Chuanxiong, Honghua, and Taoren in a ratio of 120:6:5:3:3:3	ox-LDL-induced VMSCs	α-SMA protein and cell proliferation↓ Cell invasion and migration↓ RHOA/ROCK pathway↓	(205)
Huanglian Jiedu decoction	Huanglian, Huangqi, Huangbo, and Zhizi in a ratio of 9:6:6:9	HFD-induced ApoE^-/-^ mice	Levels of TC, TG, LDL-c↓and HDL-c↑ Expression of CRP, IL-6, TNF-α↓	(206)
Liuwei Dihuang formula	Dihuang, Shanzhuyu, Chinese Yam, Zexie, Diaozhilan, and Fuling in a ratio of 32:16:16:12:12:12	HFD-induced ApoE^-/-^ mice Hcy-induced HUVECs	HUVEC apoptosis↓ The ratio of SAM/SAH and plaque formation↓	(207)
Liuwei Dihuang soft capsule	Dihuang, Shanzhuyu, Chinese Yam, Zexie, Diaozhilan, and Fuling in a ratio of 32:16:16:12:12:12	HFD-induced ApoE^-/-^ mice PDGF-BB-induced VSMCs	Lipid deposition and levels of TG, TC, LDL-c↓ Expression of ERα, ERβ, SRC3↑ CyclinD expression and cell migration↓	(208)
Danggui Buxue decoction	Danggui and Huangqi in a ratio of 1:5	hyperplasia/neointima mice model	Levels of IL-1β, TNF-α, MCP-1↓ PI3K/Akt pathway↓	(209)
Qingre Huoxue decoction	Huangqin, Chishao, Chuanxiong, Maodongqing, Honghua, Jiangxiang, and Danshen in a ratio of 3:3:2:6:2:2:6	HFD-induced ApoE^-/-^ mice LPS-induced RAW264.7 cells	Body weight and levels of TC, TG, LDL-c↓ Plaque area↓and M2 polarization↑ NF-κB pathway↓	(210)
Liuwei Dihuang formula	Shudihuang, Shanzhuyu, Shanyao, Zexie, Mudanpi, and Fuling in a ratio of 8:4:4:3:3:3	Ang II-induced VSMCs	VSMC proliferation and migration↓ Expression of α-SMA and OPN↓	(211)
Chaihu-Shugan-San formula	Chaihu, Chenpi, Chuanxiong, Baishao, Xiangfu, Zhike, and Gancao in a ratio of 4:4:3:3:3:3:1.	HFD-induced ApoE^-/-^ mice LPS-induced HUVECs	Atherosclerotic plaque areas↓ Levels of TC, TG, LDL-c, TNF-α, IL-1β, IL-6↓ Expression of BDNF and TrkB↑	(212)
Guanmaitong granule	Huangqi, Danshen, Gualou, Huanglian, Sanqi, Xuanshen, Zhebeimu, Huzhang, Shuizhi, and Muli in a ratio of 6:3:3:1.5:3:4.5:3:2:1:0.5	HFD-induced ApoE^-/-^ mice	Levels of TG, TC, LDL-c, TNF-α, IL-6, IL-1β↓ Plaque lipid deposition↓ Plaque collagen content↓ TLR4/MyD88/NF-κB pathway↓	(213)
Myocardial ischemia-reperfusion injury				
Tongmai Yangxin pill	Dihuang, Jixueteng, Maidong, Zhiheshouwu, Ejiao, Gancao, Wuweizi, Dangshen, Cuguijia, Dazao, and Guizhi in a ratio of 10:10:6:6:6:6:6:6:4:4:2	I/R-induced myocardial injury	LVEF and LVFS↑and CK and CK-MB levels↓ MDA content and inflammatory cell infiltration↓ Cardiomyocyte apoptosis↓and PI3K/Akt pathway↑	(214)
Tongmai Yangxin pill	Dihuang, Jixueteng, Maidong, Zhiheshouwu, Ejiao, Gancao, Wuweizi, Dangshen, Cuguijia, Dazao, and Guizhi in a ratio of 10:10:6:6:6:6:6:6:4:4:2	I/R-induced myocardial injury	LVDd and LVDs↓ Inflammatory cell number↓ Activities of CK, LDH, MDA↓and NO activity↑ cAMP/PKA and NO/cGMP pathways↑	(215)
QishenYiqi dripping pill	Huangqi, Danshen, Sanqi, and Jiangxiang in a ratio of 20:65:1:33	I/R-induced myocardial injury	Myocardial infarct size, LVDd, NLRP3 expression↓ LVEF and LVFS↑and PI3K/Akt-mTOR pathway↑	(216)
Yiqi Huoxue formula	Huangqi, Danshen, Sanqi, Chuanxiong, Danggui, Yiyiren, Baizhu, Fuling, Banxia, Juhong, Dilong, and Shuizhi in a ratio of 30:15:10:10:10:15:15:15:15:10:10:3	I/R-induced myocardial injury H/R-induced H9c2 cell injury	Myocardial infarct size↓ Levels of CK and LDH↓ MDA content↓and SOD level↑ H9c2 cell proliferation↑	(217)
Huoxue Jiedu formula	Shaoyao, Chuanxiong, and Huanglian in a ratio of 1:1:1	I/R-induced myocardial injury H/R-induced H9c2 cell injury	Infarcted area, CK-MB and cTnT levels↓ Beclin-1 and LC3-II↓and Bcl-2, p62↑ PI3K/AKT/mTOR pathway↑	(218)
Dried ginger-aconite decoction	Wutou and Ginger in a ratio of 1:1	I/R-induced myocardial injury H/R-induced H9c2 cell injury	SOD level↑and MDA content↓ H9c2 cell apoptosis and myocardial infarct size↓ PI3K/AKT/GSK-3β pathway↑	(219)
Tongmai formula	Danshen, Gegen, and Chuanxiong in a ratio of 1:1:1	I/R-induced myocardial injury H/R-induced neonatal rat ventricular myocyte injury	Myocardial infarct size and cell apoptosis↓ cTnT, CK, LDH levels, and MDA content↓ GSH and SOD activities↑and ROS content↓	(220)
Xin-Ji-Er-Kang formula	Renshen, Yuzhu, Sanqi, Xiebai, Danggui, Maidong, Wuweizi, Danshen, Kushen, Gancao, Huangqi, Yinyanghuo, Jinsilian, and Bingpian in a ratio of 11.71:7.03:3.09:7.80:7.80:7.80:3.93: 7.80:7.80:7.80:11.69:7.80:7.8:0.15	I/R-induced myocardial injury H/R-induced cardiomyocyte-like cell injury	Myocardial infarct size and LVDd↓ LVEF and LVFS↑ Apoptosis of cardiomyocytes↓ JAK2/STAT3 pathway↑	(221)
Si-Miao-Yong-An decoction	Jinyinhua, Xuanshen, Danggui, and Gancao in a ratio of 5:5:3:3	I/R-induced myocardial injury	Myocardial infarct size↓and LVEF, LVFS↑ Levels of CK, LDH, TNF-α, IL-6, IL-1β↓ TLR4/NF-κB pathway↓	(222)
Heart failure				
Qishen granule	Huangqi, Danshen, Jinyinhua, Xuanshen, Fuzi, and Gancao in a ratio of 30:15:10:10:9:6	TAC-induced heart failure model TGF-β-stimulated cardiac fibroblasts	LVDd and LVDs↓and LVEF and LVFS↑ Collagen deposition↓ TGF-β/SMADs and PI3K/GSK-3β pathways↓	(223)
Si-Miao-Yong-An decoction	Rendong, Xuanshen, Danggui, and Gancao in ratio of 3:3:2:1	ISO-induced heart failure model ISO-induced H9c2 cell injury	LVEF and LVFS↑and LVDd and LVDs↓ Expression of fibronectin, collagen I, α-SMA↓ PDE5A-Akt and TLR4-NOX4 pathways↓	(224)
Lingguizhugan decoction	Fuling, Guizhi, Baizhu, and Gancao in a ratio of 4:3:3:3	TAC-induced heart failure model	LVEF and LVFS↑and LVDd and LVDs↓ Heart weight, ANP, BNP, α-MHC, cardiac fibrosis↓ Akt-GSK3β/mTOR/P70S6K pathway↓	(225)
XinLi formula	Cheqiancao, Huangqi, Hongshen, Ezhu, and Shanzhuyu in a ratio of 30:40:10:9:12	LAD-induced heart failure model Ang II-induced H9c2 cell injury	LVEF↑and levels of NT-proBNP, cTnT, CK-MB↓ Content of ALD, AGTR1, TGF-β1, HYP↓ Expression of NLRP3, caspase-1, IL-1β, IL-18↓	(226)
Zhenwu decoction	Wutou, Shaoyao, Baishu, Fuling, and Ginger in a ratio of 3:3:2:3:3	DOX-induced heart failure model	LVDd and LVDs↓and LVFS and LVEF↑ Levels of CK-MB, BNP, and NT-proBNP↓ Fibrosis area, collagen I↓and SOD activity↑ Expression of IL-1β, TNF-α, IL-6↓ NF-κB pathway↓and PI3K/Akt pathway↑	(227)
Linggui Zhugan decoction	Fuling, Guizhi, Baizhu, and Gancao in a ratio of 4:3:3:2	LAD-induced heart failure model	LVEF and LVFS↑and LVDs and LVDd↓ MDA production and NT-proBNP levels↓ SOD activity and SIRT1/AMPK/PGC1α pathway↑	(228)
Shenqi Lixin decoction	Renshen, Huangqi, Rougui, Yinyanghuo, Luhui, Shuweicao, Fuling, Baishu, Longyacao, Yimucao, and Gancao in a ratio of 4:4:2:4:3:3:4:3:6:3:2	Adriamycin-induced heart failure model	LVEF and LVFS↑and LVDs and LVDd↓ Myocardial fibrosis↓ NT-proBNP level↓and ATP level↑ Expression of Bax and caspase-3↓	(229)
Jijiu Huiyang decoction	Fuzi, Ginger, Danshen, Baizhu, Taoren, Honghua, and Zhigancao in a ratio of 5:3:9:9:6:6:5	DOX-induced heart failure model	LVEF and LVFS↑ LVDs and LVDd↓ PPARα pathway↓	(230)
Xinfuli granule	Huangqi, Renshen, Danshen, Fuling, and Maidong in a ratio of 9:6:3:3:2	LAD-induced heart failure model Hypoxia/ischemia-induced H9c2 cell injury	LVEF and LVFS↑and LVDs and LVDd↓ Levels of ADP, AMP, LA, LDH, FFA↓ RHOA/ROCK pathway↓	(231)
Qishen granule	Huangqi, Danshen, Rendong, Xuanshen, Wutou, and Gancao in a ratio of 30:15:10:10:9:6	LAD-induced heart failure model LPS-induced RAW264.7 cells	LVEF and LVFS↑and LVDs and LVDd↓ Levels of CK-MB and LDH↓ TLR4/MyD88/NF-κB pathway↓	(232)
BAOXIN granule	Huangqi, Danshen, Zelan, Gancao, Maidong, Fuling, Danggui, Zhike, Dihuang, Jiegeng, Dahuang, and Mahuang in a ratio of 20:13:10:10:10:10:7:7:7:4:4:4	TAC-induced heart failure model	Heart weight and cardiac fibrosis↓ LVEF and LVFS↑and LVDs and LVDd↓ Expression of ANP, BNP, β-MHC, IL-1β, IL-6↓ Expression of TGF-β and collagen I/III↓	(233)
Guanxining injection	Danshen and Chuanxiong in a ratio of 1:1	TAC-induced heart failure model	LVEF and pro-BNP level↑ Collagen volume fraction↓ Expression of SLC7A11, GPX4↑and FTH1↓	(234)
YiQiFuMai powder	Renshen, Maidong, and Wuweizi in a ratio of 1:3:1.5	LAD-induced heart failure model	LVEF and LVFS↑and LVDs and LVDd↓ Cardiac fibrosis and p38 MAPK/ERK_1/2_ pathway↓	(235)
Guanxinning injection	Danshen and Chuanxiong	TAC-induced heart failure model	SBP, DBP, LVDs, LVDd↓ LVEF and LVFS↑and p38/c-Fos/Mmp1 pathway↓	(236)
Qiangxin recipe	Huangqi, Chuanxiong, Fuzi, Fuling, Cheqianzi, Dangshen, Guizhi, Nvzhenzi, Tinglizi, Taoren, Taizishen, and Zhuling in a ratio of 10:5:5:5:5:5:3:5:10:5:5:5	DOX-induced heart failure model DOX-induced H9c2 cell injury	Cell viability and glucose metabolism↑ Levels of BNP and cTnl↓ LVEF↑	(237)
Xinshuitong capsule	Huangqi, Danshen, Guizhi, Zexie, and Yumixu in a ratio of 6:4:4:3:3	DOX-induced heart failure model	LVEF and LVFS↑and LVDs and LVDd↓ Levels of BNP, BUN, AST, ALT↓	(238)
WuShen decoction	Renshen, Danshen, Xuanshen, Beishashen, and Kushen in a ratio of 1:3:2:2:1	LAD-induced heart failure model	LVEF and LVFS↑and LVDs and LVDd↓ Cardiac fibrosis and infarct size↓ TGF-β1/Smad2/3 pathway↓	(239)
Hypertension				
Qingda granule	Tianma, Gouteng, Huangqin, and Lianzixin in a ratio of 12:10:6:5	Spontaneously hypertensive rats Ang II-stimulated cardiac fibroblasts	SBP, DBP, MAP↓and LVEF and LVFS↑ α-SMA, collagen III, cardia fibrosis↓ TGF-β1/Smad_2/3_ pathway↓	(240)
Danzhi Xiaoyao powder	Chaihu, Baishao, Danggui, Fuling, Baizhu, Mudanpi, Zhizi, and Gancao ina ratio of 2:2:2:2:2:1:1:1	Spontaneously hypertensive rats	SBP, DBP, MAP↓ Anxiety-like behavior↓	(241)
Guizhi decoction	Guizhi, Baishao, and Gancao in a ratio of 3:2:2	HFD-induced hypertension model	Blood pressure and collagen content↓ Expression of IL-6, IL-1β, MMP2, MMP9↓	(242)
Qingda granule	Tianma, Gouteng, Huangqin, and Lianzixin in a ratio of 12:10:5:6	Ang II-hypertension model Ang II-stimulated VSMCs	SBP, DBP, MAP, Cell viability↓ MAPK and PI3K/Akt pathways↓	(243)
Gedan Jiangya decoction	Gouteng, Danshen, Gegen, Duzhong, Xiakucao, and Niuxi in a ratio of 2:5:6:3:3:4	Spontaneously hypertensive rats	SBP and DBP↓ Expression of collagen I/III, α-SMA, IL-1β, IL-6↓NF-κB pathway↓	(244)
Zhengganxifeng decoction	Niuxi, Ludou, Longgu, Mulike, Guike, Baishao, Xuanshen, Tiandong, Chuanxiong, Maiya, Yinchenhao, and Gancao in a ratio of 30:30:15:15:15:15:15:15:6:6:6:4.5	Spontaneously hypertensive rats	SBP, DBP, MAP↓ Firmicutes to Bacteroidetes ratio↓ SCFA production↑	(245)
Qing Gan Zi Shen Tang formula	Guizhencao, Weimao, Huanglian, Nvzhen, Shanzhuyu, and Xuanshen in a ratio of 10:5:1:4:4:5	HFD-induced hypertension model	SBP, DBP, MAP↓ Levels of TG, LDL-c↓and HDL-c↑	(246)
Zi Shen Huo Luo formula	Xuanshen, Niuxi, Huanglian, Mudan, Yimucao, and Rougui in a ratio of 20:15:12:12:20:3	Spontaneously hypertensive rats Aldosterone-induced H9c2 cells and cardiac fibroblasts	SBP, DBP, MAP↓and LVSP, ± dp/dt max↑ Cardiac fibrosis↓and cell proliferation↑ EGFR/ERK pathway↓	(247)
Myocardial infarction				
Buyang Huanwu decoction	Huangqi, Danggui, Chisao, Chuanxiong, Taoren, Honghua, and Dilong in a ratio of 120:10:10:10:10:10:4.5	Ligature-induced myocardial infarction model	Angiogenesis↑ PI3K/Akt/GSK3β pathway↑	(248)
Taohong siwu decoction	Shudihuang, Chuanxiong, Chishao, Danggui, Honghua, and Taoren in a ratio of 3:2:2:3:3:4	Ligature-induced myocardial infarction model TGF-β1-induced cardiac fibroblasts	Myocardial fibrosis↓ Cell proliferation and collagen expression↓ TGFBR1/Smad2/3 pathway↓	(249)
Xuefu Zhuyu decoction	Danggui, Dihuang, Taoren, Honghua, Chisao, Zhiqiao, Gancao, Chaihu, Chuanxiong, Jiegeng, and Niuxi in a ratio of 9:9:12:9:6:6:6:3:4.5:4.5:9	Ligature-induced myocardial infarction model	Mitochondria damage↓ Number of autophagosomes and lysosomes↓ Expression of LC3-B and P62↓	(250)
Yiqihuoxue decoction	Huangqi, Danggui, Renshen, Chuanxiong, and Sanqi	Ligature-induced myocardial infarction model	LVEF and LVFS↑and levels of LDH, CK-MB↓ JNK/MAPK pathway↑	(251)
Qingre Huoxue decoction	Huangqin, Shaoyao, Chuanxiong, Maodongqing, Honghua, Jiangxiang, and Danshen in a ratio of 3:3:2:6:2:2:6	Ligature-induced myocardial infarction model	LVEF and LVFS↑ MCP-1, IL-17A, TNF-α and IL-1β levels↓ LC3B, Beclin-1, ATG5, ATG7↑and p62 level↓ PI3K/Akt pathway↓	(252)
Qingyi decoction	Dahuang, Baishao, Chaihu, Zhizi, Yanhusuo, Muxiang, and Huangqin, in a ratio of 3:3:3:3:2:2:2	Severe acute pancreatitis-induced myocardial infarction model	LVEF and LVFS↑ Levels of IL-1β, IL-6, TNF-α↓ STIM1/Orai1-SOCE pathway↓	(253)
Shuangxinfang	Danshen, Chuanxiong, Baihe, and Dazao in a ratio of 20:12:30:30	Ligature-induced myocardial infarction model	LVEF and LVFS↑and LVDs and LVDd↓ Myocardial fibrosis and levels of IL-1β, TNF-α↓ TLR4/NF-κB pathway↓	(254)
Qishen granule	Huangqi, Danshen, Rendong, Xuanshen, Wutou, and Gancao in a ratio of 30:15:10:10:9:6	Ligature-induced myocardial infarction model OGD/R, ISO, Ang II and LPS-ATP-induced H9c2 cell injury	LVEF and LVFS↑and LVDs and LVDd↓ Levels of LDH, CK-MB, NLRP3, IL-1β, IL-18↓ Cell apoptosis, ROS level, NF-κB pathway↓	(101)
Others				
Jia-Wei-Si-Miao-Yong-An decoction	Jinyinhua, Lianqiao, Xuanshen, Rougui, Danggui, Danshen, Gancao, and Huzhang in a ratio of 15:15:15:9:15:15:15:9	Acute coronary syndrome model (acute coronary syndrome)	Levels of CK-MB, cTnl, IL-2, TNF-α↓ The abundance of *Bacteroides* and *Rikenellaceae RC9 gut group*↑ The abundance of *Clostridium sensu stricto 1*, *Prevotella*, *unclassified o Bacteroidales*, and *Ruminococcus gauvreauii group*↓	(255)
Zhen-Wu decoction	Fuzi, Shaoyao, Fuling, Baizhu, and Shengjiang in a ratio of 3:3:3:2:3	Uremia-induced cardiac endothelial injury Npx-induced cardiovascular endothelial injury (uremic cardiomyopathy)	LVEF↑and fibrosis area, MDA level↓ Expression of IL-1β and IL-6↓ Cell death and ROS level↓ Nrf2/keap1 pathway↑	(256)
Qingda granule	Tianma, Gouteng, Huangqin, Hehua in a ratio of 12:10:6:5	Obesity-induced hypertension and cardiac dysfunction (hypertension and cardiac dysfunction)	SBP, DBP, MAP↓and LVEF, LVFS↑ Levels of TG, TC↓and HDL-c, Akt pathway↓	(257)
Si-Miao-Yong-An decoction	Jinyinhua, Xuanshen, Danggui, and Gancao in a ratio of 3:3:2:1	TAC-induced heart failure model (heart failure)	LVEF↑and fibrosis area and collagen content↓ TGFβ1/TAK1/p38/Smad pathway↓	(258)
Huoxin pill	Lingzhi, Linshe, Xiongzhang, Niudanfen, Zhenzhufen, Renshen, Ganchan, Chuanwutou, Bingpian, and Honghua in a ratio of 20:1.2:2.4:1.2:2.4:18:1.8:9:1.2:2	ISO-induced cardiac fibrosis model (myocardial fibrosis)	Expression of α-SMA and collagen I/III↓ Cell viability and migration↓ TGF-β1/Smad pathway↓	(259)
Yunpi-Huoxue-Sanjie formula	Baizhu, Zhiqiao, Tianhuafen, Muli, and Tubiechong in a ratio of 5:2:3:10:2	HFD/streptozotocin-induced diabetic cardiomyopathy High glucose-induced H9c2 cells (diabetic cardiomyopathy)	Levels of FFA, TG, MDA↓and CAT activity↑ LVDs and LVDd↑and LVEF and LVFS↓ Expression of Atg7, Beclin1, LC3 II/LC3 I↑	(260)
Fufang Xueshuantong formula	Sanqi, Danshen, Huangqi, and Xuanshen in a ratio of 25:8:5:8	Streptozotocin-induced diabetic cardiomyopathy (diabetic cardiomyopathy)	LVEF and LVFS↑and collagen I/III and TGF-β1↓ Wnt/β-Catenin pathway↓	(261)
Danzhi Jiangtang capsule	Taizishen, Dihuang, Mudanpi, Xieze, Tusizi, and Shuizhi in a ratio of 6:5:4:4:3:3	HFD/streptozotocin-induced diabetic cardiomyopathy High glucose-induced H9c2 cells (diabetic cardiomyopathy)	LVEF and LVFS↑ Cell apoptosis and levels of IL-1β and IL-6↓ TLR4/MyD88/NF-κB pathway↓	(262)

ABCA1, ATP-binding cassette transporter A1; ACSL4, Acyl-CoA synthetase long-chain family member 4; ApoE^-/-^, Apolipoprotein-E deficient; BA, Bile acid; CK-MB, Creatine kinase MB; COX2, Cyclooxygenase-2; cTnT, Cardiac troponin T; DBP, Diastolic blood pressure; FTH1, Ferritin heavy chain 1; GPX4, Glutathione peroxidase 4; GSH, Glutathione; HDL-c, High-density lipoprotein-cholesterol; HFD, High-fat diet; H/R, Hypoxia/reoxygenation; HUVECs, Human umbilical vein endothelial cells; ICAM-1, Intercellular adhesion molecule-1; ISO, Isoproterenol; I/R, Ischemia/reperfusion; iNOS, Inducible nitric oxide synthase; LAD, left anterior descending ligation; LDH, Lactate dehydrogenase; LDLR^-/-^, LDL receptor deficient; LDL-c, Low-density lipoprotein cholesterol; LOX-1, Lectin-like oxidized low-density lipoprotein receptor-1; LVDd, Left ventricular diastolic diameter; LVDs, Left ventricular systolic diameter; LVDP, Left ventricular diastolic pressure; LVEF, Left ventricular ejection fraction; LVFS, Left ventricular shortening fraction; LVSP, Left ventricular systolic pressure; LV Vol, Left ventricle volume; MAP, Mean arterial pressure; MCP-1, Monocyte chemoattractant protein-1; MDA, Malondialdehyde; OGD/R, Oxygen-glucose deprivation/reoxygenation; PDGF, Platelet-derived growth factor; PDE5A, Phosphodiesterase 5A; PKG I, cGMP-dependent protein kinase 1; PPARγ, Peroxisome proliferator-activated receptor gamma; SAM, S-Adenosyl methionine; SAH, S-Adenosyl homocysteine; SBP, Systolic blood pressure; SRA1, scavenger receptor A1; TAC, Transverse abdominal aortic constriction; TC, Total cholesterol; TG, Triglyceride; VCAM-1, Vascular cell adhesion molecule-1; VSMCs, Vascular smooth muscle cell. ↑ upregulated, ↓ downregulated.

**Table 2:** Summary of traditional Chinese medicine extracts in the prevention and treatment of various cardiovascular diseases from 2018-2023.

Extracts	Evaluation model	Effects and action mechanism	Ref.
Atherosclerosis			
Aqueous extracts of *Tribulus terrestris*	HFD-induced ApoE^-/-^ mice ox-LDL/FBS-induced VSMCs	Liver weight and atherosclerotic plaque size↓ VSMC proliferation and migration↓ Akt/MEK/ERK pathway↓	(263)
Aqueous extracts of *Dendrobium catenatum*	High-cholesterol diet-induced zebrafish atherosclerosis model Low shear stress-induced endothelial cell dysfunction model	Atherosclerotic plaque size and macrophage infiltration↓ Levels of TC and TG↓ MDA content↓and SOD activity↑	(264)
Ethanol extracts of *Psoralea corylifolia*	HFD-induced LDLR^-/-^ mice ox-LDL-induced HUVEC injury	Atherosclerotic lesion size and macrophage infiltration↓ Expression of VCAM-1 and ICAM-1↓and cholesterol efflux↑ PARγ-ABCA1/ABCG1 pathway↑and NF-κB pathway↓	(265)
Ethyl acetate extracts of *Caesalpinia sappan*	HFD-induced ApoE^-/-^ mice	Macrophage infiltration and atherosclerotic lesion size↓	(266)
Methanol extracts of Ophiopogonis Radix	ox-LDL-induced mouse peritoneal macrophage cells	Levels of TG and TC↓ SOD, GSH-Px activities, and ABCA1 expression↑	(267)
Ethanol extracts of *Arctium lappa*	TNF-α-induced HUVEC injury	Cell viability and expression of IL-1β, TNF-α, IL-6↓ NF-κB pathway↓	(268)
Aqueous extracts of *Eucommia ulmoides*	HFD-induced ApoE^-/-^ mice	Atherosclerotic lesion sizes and total cholesterol↓ Expression of TNF-α, IL-1β, MIF↓	(269)
Ethanol extracts of *Usnea diffracta*	HFD- and vitamin D3-induced atherosclerotic rat model	Atherosclerotic lesion sizes↓ Levels of TC, TG, LDL-c↓and HDL-c↑ AST and ALT activities and levels of TNF-α, IL-1β, MCP-1↓ TLR5/MyD88/NF-κB pathway↓	(270)
Ethanol extracts of *Ganoderma lucidum* spore	HFD-induced atherosclerotic rabbit model ox-LDL-induced THP-1 cells	Levels of TC, TG, LDL-c↓and HDL-c↑ Atherosclerotic lesion sizes and foam cell formation↓ Expression of LXRα, ABCA1 and ABCG1↑	(271)
Aqueous extracts of *Salvia miltiorrhiza*	HFD-induced ApoE^-/-^ mice ox-LDL-induced HUVECs ox-LDL-induced RAW264.7 cells	Atherosclerotic lesion sizes and levels of TG and IL-6↓ Expression of p62↓and LC3B II↑ Foam cell formation↓	(272)
Ethanol extracts of *Salvia miltiorrhiza*	HFD-induced atherosclerotic rat model	Levels of TC, TG, LDL-c↓and HDL-c↑ Abundance of *Actinobacteriota* and *Proteobacteria*↑ Growth of *Firmicutes* and *Desulfobacterita*↓	(273)
Butanol extracts of *Acanthopanax senticosus*	HFD-induced ApoE^-/-^ mice	Atherosclerotic lesion sizes↓ Levels of TC, TG, LDL-c↓and HDL-c↑ Levels of TNF-α, IL-1β, IL-6↓and NF-κB pathway↓	(274)
Ethanol extracts of *Edgeworthia gardneri*	HFD-induced ApoE^-/-^ mice ox-LDL-induced macrophages and RAW264.7 cells	Atherosclerotic lesion sizes↓ Macrophage content in atherosclerotic plaque↓ Macrophage foam cell formation↓and CYP7A11 expression↑	(275)
Ethanol extract of *Schisandrae chinensis*	HFD-induced atherosclerotic rat model	Atherosclerotic lesion sizes↓ Levels of TG, LDL-c↓and HDL-c↑and Nrf2/HO-1 pathway↑	(276)
Myocardial ischemia-reperfusion injury			
Ethyl acetate extracts of *Cinnamomi Ramulus*	I/R-induced myocardial injury	LVEF and LVFS↑and expression of IL-1β, IL-6, TNF-α↓ NLRP3/Caspase-1 pathway↓	(107)
Ethanol extracts of *Origanum majorana*	I/R-induced myocardial injury LPS-treated aorta segments	Cardiac contractility (noradrenaline and endothelin-1)↓ Expression of IL-1β, IL-6↓and SOD-1↑	(113)
Ethanol extracts of *Melissa officinalis*	I/R-induced myocardial injury	dp/dt max and dp/dt min values↑ Coronary venous effluent, collagen content, oxidative stress↓	(277)
Methanol extracts of *Galium verum*	I/R-induced myocardial injury	dp/dt max values and dp/dt min↑ Levels of TBARS, O^2-^, H_2_O_2_↓and SOD, CAT activities↑	(278)
Methanol extracts of *Allium ursinum*	I/R-induced myocardial injury	dp/dt max values, dp/dt min, SLVP, SOD, CAT activities↑ Levels of TBARS, O^2-^, H_2_O_2_↓	(279)
Ethanol extracts of *Cinnamomum zeylanicum*	I/R-induced myocardial injury	Myocardial infarct size and levels of cTnl, LDH, MDA↓ SOD, GSH, and CAT activities↑	(280)
*n*-butanol extract of *Potentilla anserina*	I/R-induced myocardial injury	Activities of GSH, SOD, CAT↑and MDA content↓ Apoptosis of cardiomyocyte↓	(281)
Methanol extracts of *Dunaliella salina*	I/R-induced myocardial injury	Myocardial infarct size, LDH level, number of neutrophils↓ dp/dt max, SLVP↑and TLR4/NF-κB pathway↓	(282)
Methanol extracts of *Taraxacum officinale*	I/R-induced myocardial injury	LDH and CK levels, myocardial infarct size↓ Activities of GSH and CAT↑	(283)
Aqueous extracts of *Crataegus persica*	I/R-induced myocardial injury in diabetic rats	Expression of Nrf2, DJ-1↑ Activities of GSH, SOD, CAT↑and MDA content↓	(284)
Ethanol extracts of *Melissa Officinalis*	I/R-induced myocardial injury	Myocardial infarct size, MDA content, LDH level↓ SOD activity↑	(285)
Ethanol extracts of *Pueraria lobata* and *Salvia miltiorrhiza*	I/R-induced myocardial injury	Myocardial infarct size and levels of CK and LDH↓ VEGFR2/ERK pathway↑	(286)
Ethanol extracts of *Salvia miltiorrhiza* and *Andrographis paniculata*	I/R-induced myocardial injury	Levels of IL-6, TNF-α, IL-1β, MCP-1, IL-33↓ NLRP3/ASC/Caspase-1 pathway↓	(287)
Heart failure			
Ethanol extracts of *Crataegus pinnatifida*	DOX-induced heart failure model	LVDs and LVDd↓and dp/dt max↑ Levels of BNP, CK-MB, IL-6, IL-1β, TNF-α↓ GSH-Px and CAT activity↑and MDA content↓	(288)
Ethanol extracts of *Ginkgo biloba*	LAD-induced heart failure model	Expression of IL-1β and TNF-α↓ LVEF and LVFS↑	(289)
Ethanol extracts of *Ophiopogon japonicus*	DOX-induced heart failure model	dp/dt max, LVEF, LVFS↑and LVDs, LVDd↓ Levels of CK-MB, LDH, AST, IL-6, IL-1β, TNF-α↓ Activities of SOD, GSH-Px, CAT↑and MDA content↓ p38 MAPK pathway↓	(290)
Alkaloid extracts of *Aconitum carmichaeli*	AAC-induced heart failure model	LVEF and LVFS↑and LVDs and LVDd↓ Levels of ANP, NT-proBNP, TNF-α↓ Expression of α-SMA and collagen I/III↓	(291)
Myocardial infarction			
Aqueous extracts of *Salvia miltiorrhiza*	LAD-induced myocardial infarction model	LVEF and LVFS↑and LVDs and LVDd↓ Levels of BNP, TNF-α, IL-1β↓ TLR4/TRAF6/NF-κB pathway↓	(292)
Ethanol extracts of *Schisandra chinensis*	ISO-induced myocardial infarction model	LDH, CK levels↓and SOD, GSH-Px, CAT activities↑ Nrf2/HO-1 pathway↑	(293)
Aqueous extracts of *Spinacia oleracea*	ISO-induced myocardial infarction model	Levels of LDH, CK-MB, IL-6, TNF-α, TC, TG↓ Activities of SOD, CAT, GSH-Px and GR↑	(294)
Aqueous extracts of *Gentianella acuta*	ISO-induced myocardial infarction model	Levels of LDH, CK, IL-6, TNF-α↓ TLR4/MyD88/NF-κB pathway↓	(295)
Methanol extracts of *Agrimonia pilosa*	ISO-induced myocardial infarction model	Levels of CK-MB, LDH, CK↓ ROS generation and MDA levels↓and SOD activity↑ PI3K/Akt pathway↑	(296)
Ethanol extracts of *Syringa pinnatifolia*	LAD-induced myocardial infarction model Hypoxia-induced H9c2 cell injury	Levels of CK-MB, LDH, and inflammatory cell infiltration↓ p53-mediated apoptotic pathway↓	(297)
Ethanol extracts of *Anchusa italica*	LAD-induced acute myocardial infarction model	LVEF and LVFS↑and LVDs and LVDd↓ Myocardial infarct size and levels of TNF-α, IL-1β, IL-6↓ PI3K/Akt/mTOR pathway↓	(298)
Hypertension			
Aqueous extracts of *Whitmania pigra*	Spontaneously hypertensive rats Ang II-induced H9c2 cells	LVEF and LVFS↑and LVDs and LVDd↓ Blood pressure↓and expression of collagen I/III, TGF-β↓ H9c2 cell viability↑and p38/JNK pathway↓	(299)
Aqueous extracts of *Momordica charantia*	High salt-induced hypertension	MAP, SBP, MDA content↓and activities of CAT and SOD↑	(300)
Ethanol extracts of *Plantago asiatica*	Spontaneously hypertensive rats	MAP, SBP, collagen deposition↓ LVEF and LVFS↑and LVDs and LVDd↓	(301)
Aqueous extracts of *Eriobotrya japonica*	Spontaneously hypertensive rats Ang II-induced H9c2 cells	LVEF and LVFS↑ GATA4-NFATc3 pathway↓	(302)
Aqueous extracts of *Chimonanthus salicifolius*	Spontaneously hypertensive rats	LDL-c, TC, TG levels↓and HDL-c level↑and ERS↓	(303)
Others			
Aqueous extracts of *Salvia miltiorrhiza*	HFD-fed db/db mice High glucose-induced VSMCs	Plaque area and ROS generation↓ Expression of KLF10 and HO-1↓and cell viability↓	(304)
Ethanol extracts of *Plantago asiatica*	ISO-cardiac hypertrophy ISO-induced H9c2 cells	Collagen deposition and expression of BNP, ANP, β-MHC↓ Cardiomyocyte apoptosis↓	(106)
Ethanol extracts of *Lycium chinense*	HFD/streptozotocin-induced diabetic cardiomyopathy	Blood glucose and levels of TG, AST, LDH, CK-MB↓ Expression of IL-6, IL-1β, TNF-α↓ MDA content↓and activities of CAT, GSH-Px, SOD↑ p53-mediated apoptotic pathway and NF-κB pathway↓	(305)
Aqueous extracts of *Arnebiae Radix*	Acetylcholine and CaCl_2_-induced atrial fibrillation	AF duration↓and induction time of AF↑ Atrial fibrosis, α-SMA, and collagen I expression↓ LVFS↑and atrial enlargement (LAD, LA area)↓	(306)
Aqueous extracts of *Dendrobium candidum*	ISO-induced cardiac hypertrophy model ISO-induced H9c2 cells	LVSP, Heart body/body weight ratio, LV/TL ratio↓ Serum levels of ANP and BNP↓ Collagen deposition and ERK pathway↓	(307)
Ethanol extracts of *Smilax glabra*	TAC-induced cardiac hypertrophy model ISO-induced H9c2 cells	Myocardial fibrosis and collagen content↓ Expression of ANP, BNP, β-MHC, NT-proBNP↓ Raf/MEK/ERK pathway↓	(308)
Ethanol extracts of *Centella asiatica*	ISO-induced cardiac hypertrophy model ISO-induced atrial cardiomyocytes	Heart/body weight ratio↓and levels of AST, BNP, ANP↓ Collagen content, cardiac fibrosis, expression of TNF-α, IL-6↓ MDA content↓and SOD expression↑ PI3K/Akt pathway↑and NF-κB pathway↓	(309)
Aqueous extracts of *Angelica sinensis* and *Hedysarum polybotrys*	X-irradiation-induced myocardial fibrosis X-irradiation-induced cardiac fibroblasts	Myocardial fibrosis↓and TGF-β1 expression↓ Cardiac fibroblast apoptosis↓ Expression of miR-21, collagen 1α, c-Jun, OPN↓	(310)
Aqueous extracts of *Salvia miltiorrhiza* and *Carthamus tinctorius*	HFD/streptozotocin-induced diabetic cardiomyopathy Sodium palmitate-treated H9c2 cells	Glucose level↓and insulin level↑ Cardiomyocyte cross-sectional↓and LVFS↑ Levels of BNP and cell apoptosis↓	(311)

AAC, Abdominal aortic coarctation surgery; ANP, Atrial natriuretic peptide; BNP, Brain natriuretic peptide; dp/dt min, Minimum rate of left ventricular pressure development; dp/dt max, Maximum rate of left ventricular pressure development; GSH, glutathione; LA, left atrium; LAD, Left atrial diameter; LVEDP, Left ventricular end-diastolic pressure; LV/TL, Left ventricular weight/tibia length; LVSP, Left ventricular systolic pressure; SLVP, Systolic left ventricular pressure. ↑ upregulated, ↓ downregulated.

**Table 3:** Summary of traditional Chinese medicine compounds in the prevention and treatment of various cardiovascular diseases from 2018-2023.

Compound	cardiovascular diseases (model)	Biological activity	Ref.
Phenolic acids			
Salvianolic acid A	Atherosclerosis (animal and cellular models)	Anti-pyroptosis and anti-inflammation	(312)
	Myocardial infarction (animal and cellular models)	Anti-apoptosis	(313)
	Diabetic cardiomyopathy (animal model)	Improving mitochondrial function and anti-apoptosis	(314)
	Hypertension (animal and cellular models)	Anti-apoptosis	(315)
Salvianolic acid B	Atherosclerosis (cellular model)	Anti-inflammation, anti-pyroptosis, and anti-ERS	(316)
	Myocardial ischemia-reperfusion injury (animal and cellular models)	Anti-ferroptosis, anti-apoptosis, antioxidant, and anti-inflammation	(317, 318)
	Myocardial infarction (animal model)	Anti-ferroptosis	(119)
	Uremic cardiomyopathy (animal model)	Anti-inflammation and anti-fibrosis	(319)
	Diabetic cardiomyopathy (animal and cellular models)	Angiogenesis	(320)
Chlorogenic acid	Heart failure (animal model)	Anti-inflammation, antioxidant, and anti-apoptosis	(321)
	Myocardial infarction (animal model)	Anti-inflammation and anti-oxidative stress	(322)
	Hypertension (animal model)	Modulation of gut microbiota	(323)
	Diabetic cardiomyopathy (animal and cellular models)	Anti-ERS and anti-apoptosis	(324)
Gallic acid	Atherosclerosis (animal model)	Modulation of gut microbiota	(325)
	Heart failure (animal and cellular models)	Activation of autophagy and anti-fibrosis	(326, 327)
	Atrial fibrillation (animal model)	Inhibiting immunoproteasome	(328)
	Hypertension (animal model)	Antioxidant	(329)
	Cardiac hypertrophy (animal model)	Antioxidant	(330)
Syringic acid	Myocardial ischemia-reperfusion injury (animal model)	Anti-apoptosis	(331)
	Cardiac hypertrophy (animal model)	Anti-fibrosis	(332)
	Diabetic cardiomyopathy (animal model)	Antioxidant	(333)
Caffeic acid	Atherosclerosis (animal model)	Anti-inflammation	(334)
	Hypertension (animal model)	Antioxidant	(335)
	Cardiac remodeling (animal and cellular models)	Anti-fibrosis	(336)
Punicalagin	Atherosclerosis (cellular model)	Anti-inflammation	(337)
	Myocardial ischemia-reperfusion injury (animal model)	Antioxidant and anti-apoptosis	(338)
	Diabetic cardiomyopathy (animal and cellular models)	Improving mitochondrial function	(339)
Ferulic acid	Atherosclerosis (animal model)	Modulation of gut microbiota	(120)
	Myocardial ischemia-reperfusion injury (animal model)	Anti-ferroptosis and antioxidant	(340)
	Heart failure (animal model)	Antioxidant and anti-apoptosis	(341)
	Myocardial infarction (cellular model)	Activation of autophagy	(342)
	Diabetic cardiomyopathy (animal model)	Modulation of gut microbiota and anti-apoptosis	(343)
Cinnamic acid	Atherosclerosis (animal model)	Antioxidant	(344)
	Myocardial ischemia-reperfusion injury (animal model)	Anti-inflammation and anti-pyroptosis	(118)
	Cardiomyopathy (animal and cellular models)	Antioxidant, anti-inflammation, and anti-dyslipidemia	(345, 346)
Flavonoids			
Formononetin	Atherosclerosis (cellular model)	Anti-inflammation and antioxidant	(347)
	Myocardial ischemia-reperfusion injury (animal model)	Anti-inflammation and antioxidant	(348)
	Myocardial infarction (animal model)	Anti-inflammation	(349)
	Hypertension (animal model)	Anti-inflammation	(350)
Baicalein	Atherosclerosis (cellular model)	Anti-inflammation	(128)
	Myocardial ischemia-reperfusion injury (cellular model)	Antioxidant	(351)
	Hypertension (cellular model)	Anti-fibrosis and anti-inflammation	(352)
	Cardiac hypertrophy (animal model)	Antioxidant and activation of autophagy	(353)
	Diabetic cardiomyopathy (animal model)	Antioxidant and anti-inflammation	(354)
Baicalin	Atherosclerosis (animal model)	Anti-inflammation	(355)
	Myocardial ischemia-reperfusion injury (animal and cellular models)	Anti-ferroptosis and anti-inflammation	(356, 357)
	Cardiac hypertrophy (animal model)	Activation of the SIRT3 pathway	(358)
	Cardiomyopathy (animal model)	Anti-inflammation	(359)
	Hypertension (animal model)	Modulation of gut microbiota	(360)
Hesperidin	Atherosclerosis (animal model)	Anti-inflammation	(361)
	Myocardial ischemia-reperfusion injury (animal model)	Inhibition of autophagy	(362)
	Cardiac hypertrophy (animal model)	Anti-inflammation, anti-apoptosis, and antioxidant	(363)
Hyperoside	Atherosclerosis (cellular model)	Anti-inflammation	(364)
	Myocardial ischemia-reperfusion injury (animal model)	Antioxidant	(365)
	Myocardial infarction (animal model)	Anti-inflammation	(366)
	Heart failure (animal model)	Anti-apoptosis and activation of autophagy	(367)
Puerarin	Atherosclerosis (cellular model)	Anti-inflammation and antioxidant	(368)
	Myocardial ischemia-reperfusion injury (animal and cellular models)	Anti-ferroptosis and anti-inflammation	(369)
	Heart failure (animal and cellular models)	Anti-apoptosis and anti-inflammation	(370)
	Cardiac hypertrophy (animal model)	Activation of PPARα/PGC-1 pathway	(371)
	Hypertension (animal model)	Antioxidant	(372)
	Myocardial infarction (animal model)	Anti-apoptosis	(373)
	Diabetic cardiomyopathy (animal and cellular models)	Anti-inflammation	(374)
Quercetin	Atherosclerosis (cellular model)	Anti-inflammation and activation of autophagy	(375)
	Myocardial ischemia-reperfusion injury (animal and cellular models)	Anti-apoptosis	(376)
	Diabetic cardiomyopathy (animal model)	Anti-inflammation	(377)
	Myocardial infarction (animal model)	Anti-fibrosis	(378)
	Atrial fibrillation (animal and cellular models)	Anti-fibrosis	(379)
Kaempferol	Atherosclerosis (animal model)	Antioxidant	(380)
	Heart failure (animal model)	Antioxidant and anti-inflammation	(381)
	Diabetic cardiomyopathy (animal model)	Antioxidant	(382)
Naringenin	Atherosclerosis (animal model)	Anti-inflammation, activation of autophagy, and anti-ERS	(383, 384)
	Myocardial ischemia-reperfusion injury (animal and cellular models)	Anti-ferroptosis, antioxidant, and anti-inflammation	(130, 385)
	Hypertension (animal model)	Antioxidant	(131)
	Cardiac hypertrophy (animal and cellular models)	Antioxidant	(386)
	Diabetic cardiomyopathy (animal model)	Antioxidant, anti-inflammation, and anti-apoptosis	(387)
Tilianin	Atherosclerosis (cellular model)	Anti-inflammation	(388)
	Myocardial ischemia-reperfusion injury (animal model)	Antioxidant, anti-apoptosis, and anti-inflammation	(389, 390)
	Diabetic cardiomyopathy (animal and cellular models)	Antioxidant and anti-inflammation	(391)
Biochanin A	Atherosclerosis (animal and cellular models)	Anti-inflammation	(392)
	Myocardial ischemia-reperfusion injury (animal model)	Anti-inflammation	(393)
	Diabetic cardiomyopathy (animal model)	Antioxidant	(394)
	Myocardial infarction (animal model)	Anti-inflammation	(395)
Hydroxysafflor Yellow A	Atherosclerosis (animal model)	Anti-inflammation	(396)
	Myocardial ischemia-reperfusion injury (animal model)	Activation of autophagy and anti-inflammation	(397)
	Diabetic cardiomyopathy (animal model)	Antioxidant	(398)
	Cardiac hypertrophy (animal model)	Antioxidant	(399)
Xanthohumol	Atherosclerosis (cellular model)	Modulation lipid metabolism	(400)
	Myocardial ischemia-reperfusion injury (animal model)	Anti-ferroptosis	(401)
	Cardiac hypertrophy (animal model)	Anti-fibrosis	(402)
Dihydromyricetin	Atherosclerosis (animal model)	Anti-inflammation	(403)
	Myocardial ischemia-reperfusion injury (animal and cellular models)	Improving mitochondrial function and antioxidant	(404)
	Cardiomyopathy (animal model)	Anti-inflammation and antioxidant	(405)
	Cardiac hypertrophy (animal model)	Antioxidant	(406)
Acacetin	Atherosclerosis (animal model)	Antioxidant and anti-inflammation	(407)
	Myocardial ischemia-reperfusion injury (animal model)	Antioxidant, anti-inflammation, and anti-apoptosis	(408)
	Cardiac hypertrophy (animal model)	Anti-inflammation, antioxidant, and anti-apoptosis	(409)
	Diabetic cardiomyopathy (animal and cellular models)	Antioxidant	(410)
	Hypertension (animal model)	Improving mitochondrial function	(411)
Icariin	Atherosclerosis (animal and cellular models)	Anti-apoptosis	(412)
	Myocardial ischemia-reperfusion injury (cellular model)	Antioxidant and anti-ferroptosis	(413)
	Myocardial infarction (animal model)	Immunomodulatory	(414)
	Atrial fibrillation (animal model)	Improving mitochondrial function	(415)
	Hypertension (animal model)	Antioxidant	(416)
	Cardiac hypertrophy (cellular model)	Activation of autophagy	(417)
	Diabetic cardiomyopathy (animal model)	Improving mitochondrial function and anti-fibrosis	(418)
Scutellarin	Atherosclerosis (animal model)	Anti-apoptosis	(125)
	Myocardial ischemia-reperfusion injury (animal and cellular models)	Anti-inflammation and anti-apoptosis	(126)
	Cardiac hypertrophy (cellular model)	Anti-inflammation	(123)
	Diabetic cardiomyopathy (animal model)	Anti-apoptosis, anti-inflammation, and antioxidant	(124, 419)
	Myocardial infarction (animal model)	Antioxidant, anti-apoptosis, and anti-inflammation	(127)
Morin	Atherosclerosis (cellular model)	Anti-inflammation and activation of autophagy	(420)
	Myocardial ischemia-reperfusion injury (animal model)	Antioxidant	(421)
Epigallocatechin-3-gallate	Myocardial ischemia-reperfusion injury (animal model)	Antioxidant and anti-inflammation	(422)
	Heart failure (animal model)	Antioxidant	(423)
	Myocardial infarction (animal model)	Anti-apoptosis and anti-inflammation	(424)
	Hypertension (animal model)	Antioxidant	(425)
	Cardiac hypertrophy (cellular model)	Improving mitochondrial function and anti-fibrosis	(426, 427)
	Diabetic cardiomyopathy (animal model)	Anti-fibrosis	(428)
	Atrial fibrillation (animal model)	Anti-fibrosis	(429)
Stilbenes			
Resveratrol	Atherosclerosis (cellular model)	Anti-inflammation	(430)
	Myocardial ischemia-reperfusion injury (cellular model)	Anti-ferroptosis, improving mitochondrial function, and antioxidant	(134, 431)
	Heart failure (patients with heart failure)	Anti-inflammation	(432)
	Myocardial infarction (animal model)	Antioxidant, anti-inflammation, and anti-ferroptosis	(433, 434)
	Hypertension (animal model)	Antioxidant, anti-inflammation, and modulation of gut microbiota	(435, 436)
	Cardiac hypertrophy (animal model)	Antioxidant and activation of autophagy	(437)
	Diabetic cardiomyopathy (animal model)	Antioxidant	(438)
	Atrial fibrillation (animal model)	Anti-apoptosis and anti-fibrosis	(439)
Polydatin	Atherosclerosis (animal model)	Anti-inflammation, antioxidant, and activation of autophagy	(138, 440)
	Myocardial infarction (cellular model)	Antioxidant	(137)
	Cardiomyopathy (animal model)	Improving mitochondrial function and antioxidant	(441)
Raloxifene	Atherosclerosis (animal model)	Anti-inflammation	(442)
	Heart failure (animal model)	Anti-inflammation and antioxidant	(443)
Anthraquinones			
Emodin	Myocardial ischemia-reperfusion injury (cellular model)	Anti-inflammation and anti-pyroptosis	(444)
	Heart failure (animal model)	Anti-apoptosis	(445)
	Cardiac hypertrophy (animal model)	Anti-fibrosis	(446)
Aloe-emodin	Atherosclerosis (animal model)	Activation of autophagy	(150)
	Myocardial infarction (animal model)	Anti-apoptosis and anti-fibrosis	(151)
	Hypertension (animal and cellular models)	Anti-inflammation	(152)
Kanglexin	Atherosclerosis (animal and cellular models)	Hypolipidemic	(447)
	Myocardial ischemia-reperfusion injury (animal model)	Anti-inflammation and anti-pyroptosis	(448)
Saponins			
Astragaloside IV	Atherosclerosis (cellular model)	Anti-inflammation, antioxidant, and anti-apoptosis	(157, 449)
	Myocardial ischemia-reperfusion injury (animal model)	Antioxidant and anti-apoptosis	(450)
	Heart failure (animal model)	Angiogenesis	(451)
	Myocardial infarction (animal and cellular models)	Anti-inflammation, angiogenesis, and anti-pyroptosis	(155, 452)
	Hypertension (animal model)	Anti-inflammatory and antioxidant	(453)
	Diabetic cardiomyopathy (animal model)	Anti-ferroptosis, antioxidant, and activation of autophagy	(454, 455)
Ginsenoside Rb1	Atherosclerosis (cellular model)	Antioxidant and anti-inflammation	(456)
	Myocardial ischemia-reperfusion injury (animal and cellular models)	Antioxidant and improving mitochondrial function	(457)
	Heart failure (animal model)	Improving mitochondrial function	(458)
	Diabetic cardiomyopathy (animal model)	Antioxidant, anti-apoptosis, anti-fibrosis, and anti-inflammation	(459)
Ginsenoside Rb2	Atherosclerosis (animal and cellular models)	Anti-inflammation	(460)
	Myocardial ischemia-reperfusion injury (animal model)	Anti-inflammation and antioxidant	(461)
Notoginsenoside R1	Atherosclerosis (cellular model)	Anti-inflammation, anti-apoptosis, and antioxidant	(462)
	Myocardial ischemia-reperfusion injury (animal model)	Anti-apoptosis	(463)
	Cardiomyopathy (animal and cellular models)	Anti-apoptosis, antioxidant, and anti-fibrosis	(464)
	Cardiac hypertrophy (animal model)	Anti-inflammation	(465)
Terpenoids			
Tanshinone IIA	Atherosclerosis (animal model)	Anti-inflammation and anti-pyroptosis	(466)
	Myocardial ischemia-reperfusion injury (animal model)	Antioxidant, anti-inflammation, and anti-apoptosis	(467)
	Myocardial infarction (animal model)	Antioxidant	(468)
	Diabetic cardiomyopathy (cellular model)	Anti-ERS and anti-oxidative stress	(469)
	Cardiac fibrosis (animal model)	Anti-fibrosis and antioxidant	(470)
Paeoniflorin	Atherosclerosis (cellular model)	Anti-apoptosis and activation of autophagy	(163)
	Myocardial ischemia-reperfusion injury (animal model)	Antioxidant and anti-apoptosis	(471)
	Heart failure (animal model)	Anti-fibrosis	(472)
	Hypertension (animal model)	Anti-inflammation and antioxidant	(473)
Catalpol	Atherosclerosis (cellular model)	Anti-inflammation, antioxidant, and anti-ERS	(474)
	Myocardial ischemia-reperfusion injury (animal and cellular models)	Antioxidant and anti-inflammation	(475)
	Hypertension (cellular model)	Anti-inflammation	(476)
	Diabetic cardiomyopathy (animal model)	Anti-apoptosis	(477)
Crocin	Atherosclerosis (animal model)	Anti-inflammation	(478)
	Myocardial ischemia-reperfusion injury (animal and cellular models)	Anti-ERS	(479)
	Myocardial infarction (animal model)	Anti-inflammation	(480)
	Hypertension (animal model)	Antioxidant	(481)
	Diabetic cardiomyopathy (animal model)	Activation of autophagy and anti-apoptosis	(482)
Ginkgolide B	Atherosclerosis (animal model)	Modulation of gut microbiota, anti-inflammation, and antioxidant	(483, 484)
	Myocardial ischemia-reperfusion injury (cellular model)	Anti-inflammation and anti-apoptosis	(485, 486)
	Myocardial infarction (animal model)	Anti-inflammation	(487)
	Cardiac hypertrophy (cellular model)	Activation of autophagy	(488)
	Diabetic cardiomyopathy (animal model)	Antioxidant and anti-fibrosis	(489)
Lycopene	Atherosclerosis (animal model)	Inhibition of cholesterol and antioxidant	(490)
	Myocardial ischemia-reperfusion injury (cellular model)	Improving mitochondrial function, anti-apoptosis, and anti-ERS	(491, 492)
	Cardiac hypertrophy (animal and cellular models)	Antioxidant and improving mitochondrial function	(493)
Artemisinin	Atherosclerosis (animal model)	Anti-inflammation and antioxidant	(494, 495)
	Myocardial ischemia-reperfusion injury (animal model)	Anti-inflammation	(165)
	Hypertension (animal model)	Antioxidant	(496)
	Diabetic cardiomyopathy (animal model)	Anti-inflammation and anti-fibrosis	(497)
Oridonin	Atherosclerosis (animal model)	Anti-inflammation and antioxidant	(498)
	Myocardial ischemia-reperfusion injury (animal model)	Anti-inflammation and anti-pyroptosis	(499)
	Myocardial infarction (animal model)	Anti-inflammation and anti-fibrosis	(500)
	Cardiac hypertrophy (animal and cellular models)	Activation of autophagy	(501)
Alkaloids			
Berberine	Atherosclerosis (animal model)	Modulation of gut microbiota	(502)
	Myocardial ischemia-reperfusion injury (animal and cellular models)	Anti-inflammation, antioxidant, and anti-apoptosis	(503, 504)
	Heart failure (animal model)	Improving mitochondrial function	(505)
	Myocardial infarction (animal model)	Anti-inflammation	(506)
	Hypertension (animal model)	Modulation of gut microbiota	(507)
	Cardiac hypertrophy (animal and cellular models)	Activation of autophagy	(508)
	Diabetic cardiomyopathy (cellular model)	Anti-inflammation	(509)
Colchicine	Atherosclerosis (cellular model)	Anti-inflammation and anti-pyroptosis	(510)
	Heart failure (animal model)	Anti-inflammation	(511)
	Cardiomyopathy (animal and cellular models)	Anti-inflammation	(512)
	Myocardial infarction (animal model)	Anti-inflammation	(513)
Sinomenine	Atherosclerosis (animal model)	Anti-inflammation and antioxidant	(514)
	Myocardial ischemia-reperfusion injury (animal model)	Anti-apoptosis, anti-inflammation, antioxidant	(515)
	Heart failure (animal model)	Anti-fibrosis and anti-inflammation	(516)
	Cardiac hypertrophy (animal and cellular models)	Antioxidant and anti-inflammation	(517)
Nuciferine	Atherosclerosis (animal model)	Anti-apoptosis and activation of MMP12/Akt pathway	(518)
	Myocardial ischemia-reperfusion injury (animal model)	Anti-apoptosis and activation of PPAR-γ	(519)
	Myocardial infarction (animal model)	Anti-inflammation	(520)
Polysaccharides			
*Dendrobium huoshanense*	Atherosclerosis (zebrafish model)	Antioxidant and anti-inflammation	(521)
*Laminaria japonica*	Atherosclerosis (animal model)	Modulation of gut microbiota	(522)
*Cordyceps militaris*	Atherosclerosis (animal model)	Improving hyperlipidemia	(523)
*Undaria pinnatifida*	Atherosclerosis (animal model)	Anti-inflammation	(524)
*Cipangopaludina chinensis*	Atherosclerosis (animal model)	Modulation of gut microbiota	(525)
*Poria cocos*	Atherosclerosis (animal model)	Anti-inflammation	(526)
*Lycium barbarum*	Atherosclerosis (animal model)	Modulation of gut microbiota	(527)
	Myocardial ischemia-reperfusion injury (animal model)	Improving mitochondrial function and antioxidant	(528)
	Cardiac hypertrophy (animal model)	Anti-inflammation	(529)
*Schisandra chinensis*	Cardiac hypertrophy (animal model)	Antioxidant	(530)
*Chuanminshen violaceum*	Myocardial ischemia-reperfusion injury (animal model)	Anti-ferroptosis	(531)
*Polygonatum sibiricum*	Heart failure (animal model)	Antioxidant, anti-inflammation, and anti-apoptosis	(532)
*Astragalus membranaceus*	Heart failure (animal model)	Anti-inflammation	(533)

↑ upregulated, ↓ downregulated.

### 3.1 TCM formulas for CVD

Chinese herbal compounding (*fu fang* or prescription in Chinese) is the main form of TCM for the prevention and treatment of various diseases, which is the simultaneous application of multiple herbs to regulate the body as a whole for therapeutic purposes in clinical practice. A meta-analysis showed that the efficacy of Bushen Huoxue decoction in treating coronary heart disease was superior to conventional Western medicine (95). Bi and his colleagues (96) confirmed that Qingre Huatan formulae for the phlegm-heat-stasis syndrome pattern of coronary heart disease was safe and can effectively improve vascular endothelial function. In a randomized, multicenter, double-blind, non-inferiority trial, the results showed that treatment with the Songling Xuemaikang capsule had a well-tolerated and improved total hypertension symptom score and total cholesterol in patients with essential hypertension (97). In addition, TCM prescriptions have been shown to improve sleep disorders in patients with CVD (98). Mechanistically, the Qing-Xue-Xiao-Zhi formula can alleviate the development of atherosclerosis by blocking the TLR4/MyD88/NF-κB pathway to promote lipid efflux, reducing atherosclerotic plaques in the aorta and aortic root and serum TMAO levels, and inhibiting macrophage-mediated inflammation (99). Wu et al. (100) observed that the QiShenYiQi dripping pill can inhibit myocardial ischemia-induced ferroptosis in cardiomyocytes by reducing mitochondrial ROS levels and restoring mitochondrial function (e.g., biogenesis and dynamic homeostasis). Chen et al. (101) demonstrated that Qishen granule administration exhibited cardioprotective effects by inactivation of NF-κB/NLRP3/GSDMD pathway in myocardial infarction, as evidenced by improving cardiac function, reducing inflammatory cell infiltration and collagen deposition, as well as inhibiting NLRP3 inflammasome activation and pyroptosis. Qing-Xin-Jie-Yu granule treatment contributed to the alleviation of atherosclerosis development by regulating gut microbiota composition (that is, the relative abundance of *Turicibacter* and *Roseburia* was enhanced), increasing bile acids production, and reducing metaflammation induced by HFD (102). Zhou et al. (103) showed by a comprehensive network analysis that Shenfu injection can be used to treat coronavirus disease 2019 (COVID-19) combined with heart failure. Except for the above-mentioned TCM prescriptions, there are still numerous studies reported on the use of some classical TCM formulas for the prevention and treatment of CVD according to ancient works and the modern clinical. Herein, we summarized the pharmacological effects and molecular mechanisms of TCM prescriptions on CVD based on published studies from 2018 to 2023 and listed in **Table 1**.

### 3.2 TCM extracts for CVD

Increasing evidence has proved that single TCM extracts also possessed a protective effect against CVD except for TCM preparations mentioned above (**Table 2**). For example, a network pharmacology study showed that Schisandra extracts have the potential for therapeutic effects on atherosclerosis by regulating immune inflammation and oxidative stress (104). Recently, the key mechanisms of TCM extracts in CVD may be associated with immunomodulation, antioxidant, anti-cell death, anti-inflammatory, and gut microbiota regulation. For example, Quince extract exhibited hypolipidemic, antioxidant, anti-inflammatory, anti-thrombotic, and vascular endothelium protective effects on HFD-induced atherosclerosis (105). *Plantago asiatica* L. seeds extracts prevented isoproterenol-induced cardiac hypertrophy by restoration of autophagy and inhibition of cardiomyocyte apoptosis (106). The ethyl acetate extracts of *Cinnamomi Ramulus* protect rats from myocardial ischemia-reperfusion injury by suppression of NLRP3 inflammasome activation and pyroptosis (107). In doxorubicin-induced chronic heart failure, the combination of aqueous extracts of *Aconiti Lateralis Radix Praeparata* and *Zingiberis Rhizoma* has a better therapeutic effect than their single aqueous extracts, which may be associated with improving left ventricular function and promoting mitochondrial energy metabolism through activation of the PPARα/PGC-1α/Sirt3 pathway (108). Treatment with bay leaf extracts exhibited an anti-inflammatory effect in the rat model of myocardial infarction (109), reflected by reducing the levels of C-reactive protein and myeloperoxidase. Another study showed that aqueous extracts of *Ligustrum robustum* attenuated atherosclerosis development by modulating gut microbiota composition and metabolism, as evidenced by increased relative abundance of genus *Bifidobacterium*, and reduced serum TMAO and bile acid, as well as decreased cholesterol absorption (110). In addition, single TCM extracts used for the treatment of CVD have been shown to regulate mitochondrial homeostasis and maintain normal autophagy function, as well as have anti-ERS and anti-contractile effects. For instance, Vilella et al. (111) reported that green tea extracts ameliorated cardiomyopathy progression by improving mitochondrial function. In streptozotocin-induced diabetic atherosclerosis, Ginkgo biloba leaf extracts reduced plaque lipid deposition and serum inflammatory cytokines secretion via inhibiting ERS and mTOR-mediated autophagy (112). Granado et al. (113) proved that Marjoram extracts prevented inflammatory response, apoptosis, and oxidative stress of cardiomyocytes induced by coronary ischemia-reperfusion, as well as possessed anti-contractile effects in aorta segments. Taken together, the cardioprotective effects of single TCM extracts on various CVDs were confirmed, but its underlying mechanisms and safety need to be further explored before clinical practice.

### 3.3 Compounds isolated from TCM for CVD

With the development of pharmaceutical chemistry and pharmacology, many scholars have conducted studies on the bioactive components of TCM in recent years. It has been found that a large number of effective compounds extracted from TCM, such as phenolic acids, flavonoids, stilbenes, anthraquinones, saponins, terpenoids, alkaloids, polysaccharides, etc., all of which possessed therapeutic effects on various CVDs (**Table 3**).

#### 3.3.1 Phenolic acids

Phenolic acids are a subclass of plant phenolics that can be isolated and extracted from many traditional Chinese herbs such as *Angelica sinensis*, *Salvia miltiorrhiza*, *Cinnamomi ramulus*, *Lonicera japonica*, *Radix Paeoniae Rubra*, *Ligusticum wallichii*, *etc.* Modern pharmacological studies have confirmed that phenolic acids have a variety of biological activities, including antioxidant, anti-inflammation, anti-coagulant, and hypolipidemic (114). Of note, numerous studies have demonstrated that phenolic acids have been shown to have a therapeutic effect on CVD (115, 116). Vanillic acid, a phenolic compound extracted from *Angelica sinensis*, could alleviate hypoxia/reoxygenation-induced H9c2 cardiomyocyte injury by inhibiting cell apoptosis and oxidative stress (117). Cinnamic acid is an active phenolic acid extracted from *Cinnamomi ramulus* that has a cardioprotective effect against myocardial ischemia-reperfusion injury by inhibiting NLRP3 inflammasome-mediated inflammation and cardiomyocyte pyroptosis (118). Shen et al. (119) showed that Salvianolic acid B can effectively inhibit ferroptosis and mitochondrial oxidative stress by activation of the Nrf2 pathway, thereby attenuating myocardial infarction. Another study reported that ferulic acid ameliorated atherosclerotic injury by modulating gut microbiota and lipid metabolism (120), as evidenced by reducing the relative abundance of *Erysipelotrichaceae* and *Firmicutes* and increasing the relative abundance of *Ruminococcaceae*, as well as downregulating serum levels of total cholesterol, triglyceride, and low-density lipoprotein cholesterol and atherogenic index in HFD-fed ApoE^-/-^ mice. In addition, we summarized many phenolic acids such as caffeic acid, protocatechuic acid, chlorogenic acid, gallic acid, benzoic acid, and erucic acid for the treatment and prevention of CVD, which are listed in **Table 3**.

#### 3.3.2 Flavonoids

Flavonoids are secondary metabolites widely found in TCM and have various pharmacological activities that are beneficial to human health (121), such as antioxidant, anti-apoptosis, anti-inflammation, antitumor, *etc.* Of note, many studies have found that flavonoid compounds can play an effective protective role in the treatment of CVD (122). Functionally, scutellarin, a flavonoid compound extracted from *Erigeron breviscapus*, possessed protective effects against cardiac hypertrophy (123), diabetic cardiomyopathy (124), atherosclerosis (125), myocardial ischemia-reperfusion injury (126), and myocardial infarction (127) via inhibition of inflammation, oxidative stress, and apoptosis. Baicalein extracted from *Scutellaria baicalensis* inhibited Ang II/oxidized low-density lipoprotein-induced inflammation via inactivation of the AMPK/NF-κB pathway, thus showing anti-atherosclerotic activity (128). Wogonin, one of the main flavonoid compounds of *Scutellaria radix*, ameliorated isoproterenol-induced myocardial infarction via suppression of inflammation and oxidative stress (129). Naringenin was the main flavonoid that existed in various citrus fruits, bergamots, and tomatoes. Naringenin treatment inhibited myocardial ischemia-reperfusion-induced inflammation, lipid peroxidation, and ferroptosis by activating the Nrf2/GPX4 pathway (130). Naringenin suppressed blood pressure, cholesterol triglycerides, LDL, serum malondialdehyde (MDA), and nitric oxide, as well as increased serum superoxide dismutase and glutathione via blocking the STAT3 pathway in obesity-associated hypertension (131). Abukhalil et al. (132) reported that galangin, a natural flavonoid found in lesser galangal and honey, exerted a protective effect on diabetic cardiomyopathy by reduction of oxidative stress, inflammation, and hyperglycemia. Last but not least, pinocembrin belongs to this series of flavonoids and exerts an antioxidant effect on heart failure by activating the Nrf2/HO-1 pathway, evidenced by reducing ROS level in heart tissue and serum MDA level and improving cardiac function (133). Taken together, flavonoids possess a range of biological activities that prevent the development and progression of CVD, and their potential mechanisms are summarized in **Table 3**.

#### 3.3.3 Stilbenes

Stilbenes are compounds with a stilbene parent structure connected by a vinyl group between two benzene rings and have a typical conjugated structure. Stilbenes are widely found in TCM, including *Polygonum cuspidatum* and *Polygonum multiflorum*, and have beneficial effects on human health. Resveratrol, a main compound extracted from *Polygonum cuspidatum*, can prevent myocardial ischemia-reperfusion injury by inhibition of oxidative stress and ferroptosis (134). Maayah et al. (135) found that resveratrol treatment inhibited cardiac NLRP3 inflammasome activation and reduced inflammatory responses, and thus alleviated doxorubicin-induced cardiomyopathy. Another study showed that resveratrol protects against atherosclerosis by reducing TMAO levels and enhancing hepatic bile acid biosynthesis through the remodeling of intestinal flora (136). Polydatin, an active component in *Polygonum cuspidatum*, can ameliorate acute myocardial infarction-induced cardiac damage by inhibition of oxidative stress and cell apoptosis via activation of the Nrf2/HO-1 pathway (137). Zhang and colleagues (138) confirmed that polydatin can inhibit inflammation and pyroptosis by blocking the NLRP3/caspase-1 pathway and triggering mTOR-mediated autophagy, thereby exerting an anti-atherosclerosis effect. 2,3,4’,5-tetrahydroxystilbene 2-O-β-D-glucoside (TSG) is extracted and purified from *Polygonum multiflorum*, which can prevent the development and progression of atherosclerosis by reducing lipid accumulation and inflammation in ApoE^-/-^ mice fed with HFD (139). These results suggested that stilbenes exhibited therapeutic effects on CVD via different mechanisms (**Table 3**).

#### 3.3.4 Anthraquinones

Anthraquinones are compounds with unsaturated cyclic diketone structures and are widely found in some Chinese herbal medicines (140). Accumulating studies have shown that anthraquinones have various biological activities, including antitumor, antioxidant, and anti-inflammation (141), *etc.* Emodin (1,3,8-trihydroxy-6-methylanthraquinone), a natural anthraquinone derivative, can be extracted and purified from natural plants such as *Rhei* radix et rhizoma, *Polygoni Cuspidat*, *Polygoni multiflori*, which protects against various CVDs (142). Previous studies have demonstrated that emodin exhibited a therapeutic effect on atherosclerosis via inhibition of inflammatory response (143), suppression of PPAR-γ-mediated lipid metabolism (144) and endothelial cell apoptosis (145), reducing oxidative stress (146). Other studies found that emodin can prevent cardiac hypertrophy (147), restrict vasodilation by activation of K^+^-ATP channels (148), and inhibition of myocardial fibrosis (149). Aloe-emodin is an active ingredient in *Rheum palmatum* and *Aloe vera*, which prevents the progression of various CVDs. For example, Tang et al. (150) reported that aloe-emodin exerted an anti-atherosclerosis effect by reducing atherosclerotic plaque in the aorta and lipid accumulation and promoting endothelial autophagy. Yu et al. (151) showed that aloe-emodin inhibited the development of cardiac fibrosis and hypertrophy in rats with chronic myocardial infarction by suppressing cardiac apoptosis and oxidative stress via the inactivation of the TGF-β/Smad pathway. Another study found that aloe-emodin exhibited specific therapeutic value in hypertension-related CVD by inhibiting NLRP3 inflammasome activation (152). Moreover, other anthraquinone compounds have protective effects against CVD, which is summarized in **Table 3**.

#### 3.3.5 Saponins

Saponins are a class of glycosides with triterpenoids or steranes, which are widely found in natural plants and have been reported to have many pharmacological activities, including antitumor, anti-inflammation, anti-oxidative stress, *etc.* Importantly, previous studies have shown that saponins were shown to be effective in treating CVD (**Table 3**) (153), such as atherosclerosis, myocardial infarction, myocardial ischemia-reperfusion injury, heart failure, cardiomyopathy, and hypertension. Astragaloside IV (AS-IV) is the main active ingredient purified from *Astragalus membranaceus* and serves as an effective therapeutic agent for the treatment of CVD (154). For example, AS-IV could markedly reduce myocardial infarction-induced myocardial fibrosis, cardiac hypertrophy, and macrophage pyroptosis by inhibition of the ROS/caspase-1/GSDMD pathway (155). Yin et al. (156) showed that AS-IV protects against myocardial ischemia-reperfusion injury by suppressing cardiomyocyte apoptosis and serum cardiac troponin levels via blocking CaSR/ERK_1/2_ and the related apoptotic pathways. Another study found that AS-IV treatment suppressed inflammation, plaque area, and serum lipids in HFD-induced atherosclerosis by blocking the MAPK/NF-κB pathway (157). Other studies proved that AS-IV can attenuate the progression of myocardial fibrosis (158), heart failure (159), and cardiac hypertrophy (160) by inhibiting Nrf2-mediated oxidative stress. Ginsenosides (mainly including the ginsenosides Rb1, Rb2, Rb3, Rc, Rd, Re, Rg3, and Rh2 and compound K) serve as the main active constituents of *Panax ginseng* and exert protection against CVD by suppression of oxidative stress, cholesterol accumulation, inflammation, and insulin resistance (161).

#### 3.3.6 Terpenoids

Terpenoids are a large group of organic compounds present in TCM and can be effectively used for treating various diseases. Importantly, the preventive and therapeutic effects of terpenoids on CVD have received increasing attention (**Table 3**), which was associated with their remarkable biological activities, such as anti-inflammation, antioxidant, and anti-apoptosis. Tanshinone IIA, a fat-soluble component of *Salvia miltiorrhiza*, could protect against heart failure by inhibition of cardiomyocyte apoptosis via activating the AMPK/mTOR-mediated autophagy pathway (162). Paeoniflorin, a bioactive component extracted from *Paeonia lactiflora*, can ameliorate ox-LDL-induced atherosclerosis by inhibiting apoptosis and adhesion molecule expression via autophagy enhancement in human umbilical vein endothelial cells (163). Andrographolide, a bioactive labdane diterpenoid extracted from *Andrographis paniculate*, exhibited anti-oxidative stress capacity against adverse cardiac remodeling after myocardial infarction by activating the Nrf2/HO-1 pathway (164). Artemisinin, a sesquiterpene lactone compound with peroxisome bridging group structure purified from *Artemisia annua*, prevented myocardial ischemia-reperfusion injury by inhibition of cardiac autophagy and NLRP3 inflammasome activation (165). Taken together, terpenoids may serve as an effective therapeutic agent for the treatment of various CVDs by different mechanisms.

#### 3.3.7 Alkaloids

Alkaloids are a class of nitrogen-containing basic organic compounds and widely found in TCM. Of note, alkaloids exert protective effects against CVDs by suppression of inflammation, oxidative stress, and cardiomyocyte apoptosis (**Table 3**). Berberine, a natural isoquinoline alkaloid isolated from *Rhizoma coptidis*, possessed profound pharmacological activities for the treatment of various CVDs (166), including atherosclerosis, cardiac hypertrophy, heart failure, myocardial infarction, and arrhythmia. Similarly, palmatine was a potential candidate drug for the treatment of cardiac hypertrophy by activating the Nrf2/ARE pathway (167). Matrine, a quinolizidine alkaloid derived from *Sophora flavescens*, could attenuate diabetic cardiomyopathy by reducing inflammatory cytokines levels and oxidative stress (168). Cyclovirobuxine D, a steroidal alkaloid extracted from *Buxus microphylla*, exerted a cytoprotective effect against HFD diet- and streptozotocin-induced rat diabetic cardiomyopathy by activating Nrf2-mediated antioxidant responses (169). Cordycepin is an active ingredient in *Cordyceps sinensis* that can prevent myocardial ischemia-reperfusion injury by activating the AMPK/mTOR-mediated autophagy (170). Colchicine, a botanical alkaloid derived from *Colchicum autumnale*, exerted unique anti-inflammatory effects in the therapy of various CVDs (171), including atherosclerosis, heart failure, atrial fibrillation, and myocardial infarction.

#### 3.3.8 Polysaccharides

Polysaccharides widely exist in natural plants, which are a kind of complex structure of natural polymer compounds (172). Currently, natural polysaccharides are attracting considerable attention worldwide due to their versatile biological activities and few side effects. Of note, numerous studies have shown that bioactive polysaccharides exhibit profound efficiency in controlling the risk factors of CVD (173), such as inflammatory response, oxidative stress, hypertension, and hyperlipidemia. Polysaccharides derived from *Gelidium crinale* reduced oxidative stress and inflammation in oxidized low-density lipoprotein-induced atherosclerosis (174). Huang et al. (175) found that the administration of polysaccharides from *Eriobotrya japonica* effectively reduced oxidative damage and inflammation induced by myocardial ischemia-reperfusion injury. Astragalus polysaccharides could ameliorate diabetic cardiomyopathy progression by improving cardiac function and inhibiting cardiomyocyte apoptosis via the inactivation of the ERS pathway (176). *Lycium barbarum* polysaccharides could reduce the levels of inflammatory cytokines (e.g., IL-6 and TNF-α) and plasma lipid peroxidation in a pressure overload-induced heart failure rat model (177). In addition, polysaccharides extracted from TCM, such as *Polygonatum sibiricum*, *Opuntia dilleniid*, *Plantago asiatica*, *Angelica sinensis*, and *Ganoderma lucidum*, also have therapeutic effects on various CVDs (**Table 3**).

#### 3.3.9 Others

In addition to the above-mentioned compounds isolated from TCM for the prevention of CVD, other active ingredients in TCM have been reported to have therapeutic effects on various CVDs. Schisandrin B, bioactive dibenzocyclooctadiene derivatives found in *Schisandra chinensis*, could alleviate diabetic cardiomyopathy by reducing cardiac inflammation and damage via blocking MyD88-dependent inflammation (178). Schisandrin B prevented hypoxia/reoxygenation-induced cardiomyocyte injury by inhibiting inflammation and oxidative stress, which was associated with the activation of the AMPK/Nrf2 pathway (179). Morronisid, an iridoid glycoside extracted from *Cornus officinalis*, promoted angiogenesis and improved cardiac function in rats with acute myocardial infarction (180). Sulforaphane is a natural glucosinolate found in *Raphanus sativus*, which inhibited cardiac cell ferroptosis by activating the AMPK/Nrf2 pathway (76). Schisandrol A, a bioactive lignan extracted from *Schisandra chinensis*, could inhibit cardiomyocyte apoptosis induced by myocardial ischemia-reperfusion via increasing 14-3-3θ expression (181). Collectively, natural compounds from TCM exert anti-CVD effects, which may be developed as an effective therapeutic agent for the treatment of CVD in clinical.

## 4 Clinical study of the TCM for the prevention and treatment of CVD

Accumulating evidence has reported that TCM has a wide range of pharmacological effects in various CVDs and its beneficial efficacy has been proved *in vitro* cell models or animal experiments. Importantly, several clinical studies are underway to explore the safety and efficacy of TCM decoction and injections for the treatment of various CVDs. For example, several studies provided a reliable evaluation of the efficacy and safety of Xuefu Zhuyu granules (182) and Xuefu Zhuyu granules (183) in the treatment of patients with coronary heart disease. Other randomized controlled trials similarly analyzed the efficacy and safety of Zhuling decoction (184) and Buyang Huanwu decoction (185) in the treatment of heart failure. A multicenter, randomized, double-blind, placebo-controlled clinical trial found that Qing-Xin-Jie-Yu granule reduced inflammation and cardiovascular endpoint in patients with coronary heart disease (186). A phase I clinical trial by Hu et al. (187) showed that Danhong injection promoted endothelial progenitor cell mobilization by increasing the expression of Akt, eNOS, and MMP-9 in patients with coronary heart disease. Lai et al. (97) found that treatment with TCM formula (Songling Xuemaikang capsule) improved blood pressure in patients with mild hypertension and was well tolerated. Another study confirmed that astragalus injection was a safe and effective therapeutic agent in the clinical management of heart failure (188). In addition, several clinical trials have shown that the combination of TCM and standard drugs for CVD treatment was advantageous to simple conventional Western medicine in relieving clinical symptoms (25, 189). Chao et al. (190) reported that TCM formula combined with Western medicine reduced blood lipid levels and inflammatory factors in patients with coronary heart disease. Zhang et al. (191) showed that modified Xiaojianzhong decoction combined with conventional Western medicine alleviated the progression of chronic heart failure by improving heart function and maintaining gastrointestinal hormones. Another study found that treatment with Jianpi Huazhi pill combined with Western medicine (anti-heart failure) led to decreasing the levels of inflammatory cytokines and improving the composition of the gut microbiota (192). Meanwhile, several clinical studies are completed or ongoing to evaluate the safety and efficacy of TCM combined with Western medicine for the treatment of CVD according to Chinese Clinical Trial Registry (**Table 4**). Many researchers have proved that treatment with TCM based on the standard drug not only prevented CVD progression and improved quality of life but also reduced the incidence of adverse cardiovascular events in patients (193–195). More interestingly, TCM may be an effective alternative method to Western medicine in modern American healthcare, but some barriers prevent its integration into Western health systems, such as the fact that TCM is not accredited by the American Board of Medical Specialties, available TCM therapies may impose an undesired burden for patients, and TCM therapies are individualized. However, no cardiovascular drug or combination of drugs has shown significant efficacy in all patients with CVD, and standard Western medicine can lead to adverse side effects. From an economic point of view, TCM therapies are cheaper than Western medicine and have a better prognosis for patients with CVD. Based on the current situation, TCM may be an attractive alternative for patients with CVD.

**Table 4:** The ongoing clinical trials of traditional Chinese medicine combined with Western medicine for cardiovascular diseases therapy from 2018-2023.

No.	Disease	Interventions	Status	Sponsor	Clinical Trial ID
1	Atherosclerosis	Tongxinluo capsule+CWM	Completed	Qilu Hospital of Shandong University	ChiCTR1900025842
2	Atherosclerosis	Xiaochaihu decoction+CWM	Not recruiting	Shanghai Sixth People’s Hospital	ChiCTR2000032470
3	Atherosclerosis	Yanshi Jiangzhi formula+CWM	Not recruiting	Shanghai Tenth People’s Hospital	ChiCTR2000036785
4	Atherosclerosis	Yishen Huazhuo decoction+CWM	Not recruiting	Longhua Hospital Shanghai University of Traditional Chinese Medicine	ChiCTR2300071014
5	Atherosclerosis	Huoxue Jiedu formula+CWM	Recruiting	Xiyuan Hospital, Chinese Academy of Traditional Chinese Medicine	ChiCTR2300074283
6	Atherosclerosis	Huazhuo Tiaozhi granule+CWM	Not recruiting	Guang’anmen Hospital, China Academy of Chinese Medical Sciences	ChiCTR2400079454
7	Myocardial ischemia-reperfusion injury	Shenxiang Suhe pill+CWM	Recruiting	Sir Run Run Shaw Hospital, College of Medicine, Zhejiang University	ChiCTR2200055170
8	Heart failure	Yiqihuoxuelishui formula+CWM	Recruiting	Dongfang Hospital Affiliated to Beijing University of Chinese Medicine	ChiCTR1900022036
9	Heart failure	Yangyin Shuxin formula+CWM	Completed	The First Affiliated Hospital of Tianjin University of Traditional Chinese Medicine	ChiCTR2000030921
10	Heart failure	LuHong formula+CWM	Not recruiting	Shuguang Hospital Affiliated to Shanghai University of traditional Chinese Medicine	ChiCTR2000037368
11	Heart failure	Qiangxin formula+CWM	Recruiting	Shanghai Hospital of Traditional Chinese Medicine	ChiCTR2000037254
12	Heart failure	Shenfu Xiangshao decoction+CWM	Not recruiting	Shanghai Putuo District Central Hospital	ChiCTR2000036639
13	Heart failure	Shen’ge formula+CWM	Not recruiting	Longhua Hospital affiliated to Shanghai University of Traditional Chinese Medicine	ChiCTR2000036533
14	Heart failure	Shenshao pill+CWM	Recruiting	The First Teaching Hospital of Tianjin University of Traditional Chinese Medicine	ChiCTR2100042242
15	Heart failure	Shenge powder+CWM	Not recruiting	Nanxiang Hospital	ChiCTR2100049790
16	Heart failure	Yixin formula+CWM	Not recruiting	Yueyang Hospital of Integrated Traditional Chinese and Western Medicine Affiliated to Shanghai University of Traditional Chinese Medicine	ChiCTR2100051882
17	Heart failure	Fangji Huangqi decoction+CWM	Recruiting	The Second Affiliated Hospital of Tianjin University of Traditional Chinese Medicine	ChiCTR2100054580
18	Heart failure	Xin-Li-Fang formula+CWM	Not recruiting	The Second Affiliated Hospital of Guangzhou University of Chinese Medicine (Guangdong Provincial of Chinese Medicine)	ChiCTR2200058649
19	Heart failure	Kangxin formula+CWM	Not recruiting	The First Affiliated Hospital of Guangzhou University of Chinese Medicine	ChiCTR2300069435
20	Heart failure	Yangxinxue granules+CWM	Not recruiting	Qionglai Hospital of Traditional Chinese Medicin	ChiCTR2300074840
21	Heart failure	Shexiang Baoxin pill+CWM	Not recruiting	Sichuan Provincial People’s Hospital	ChiCTR2300076014
22	Heart failure	Yiqi Huayu decoction+CWM	Recruiting	Shuguang Hospital Affiliated to Shanghai University of Traditional Chinese Medicine	ChiCTR2400082425
23	Heart failure	Qiwei Fangji Huangqi granule+CWM	Not recruiting	Hangzhou Traditional Chinese Medicine Hospital	ChiCTR2400080029
24	Hypertension	Bushen Jiangya granule+CWM	Recruiting	Guang′anmen Hospital, China Academy of Chinese Medical Sciences	ChiCTR1900028572
25	Hypertension	Shugan Wendan decoction+CWM	Not recruiting	Guangzhou University of Chinese Medicine	ChiCTR2000034557
26	Hypertension	Dingxuan Shuyu formula+CWM	Completed	Shuguang Hospital Affiliated to Shanghai University of Chinese Medicine	ChiCTR2000040386
27	Hypertension	Chaigui decoction+CWM	Completed	Wuxi Hospital of Traditional Chinese Medicine	ChiCTR2300076783
28	Hypertension	Huoxue Qiyang Qutan prescription+CWM	Recruiting	Shanghai Yueyang Integrated Traditional Chinese Medicine and Western Medicine Hospital	ChiCTR2400081580
29	Myocardial infarction	Qishen Yiqi drop pill+CWM	Not recruiting	The Second Affiliated Hospital of Tianjin University of Traditional Chinese Medicine	ChiCTR2000029136
30				Peking University First Hospital	ChiCTR2300069035
31	Myocardial infarction	Shexiang Tongxin drop pill+CWM	Recruiting	Beijing University of Chinese Medicine Dongzhimen Hospital	ChiCTR2300075069
32	Septic cardiomyopathy	Fuling Sini decoction+CWM	Recruiting	Beijing University of Chinese Medicine Shenzhen Hospital (Longgang)	ChiCTR2100045549
33	Combined blood stasis with dilated cardiomyopathy	Kuoxinfang granule+CWM	Recruiting	Longhua Hospital, Shanghai University of Traditional Chinese Medicine	ChiCTR2100049536
34	Coronary artery disease	Shexiang Baoxin pill+CWM	Recruiting	Gansu Provincial Hospital	ChiCTR2400080152

CWM, conventional Western medicine.

## 5 Conclusion and prospects

As the leading cause of death after malignant tumors, CVD is difficult to treat clinically and imposes a huge economic and health burden on people worldwide. The morbidity and mortality of CVD are continuously increasing, and the treatment is ineffective because of its complex pathogenesis. In recent years, TCM has been particularly prominent in the treatment of 95 certain diseases, including CVD, offering a new perspective in the modern era for the prevention and treatment of diseases such as COVID-19. In this review, we found that TCM (formulas, extracts, and compounds) can combat CVD through multiple mechanisms, including anti-inflammatory, antioxidant, improving mitochondrial dysfunction, anti-cell death (such as autophagy, apoptosis, ferroptosis, pyroptosis), and regulating gut microbiota. Meanwhile, clinical trials have proven the efficacy and safety of TCM in alleviating the symptoms of CVD. However, there are still some challenges that must be overcome in TCM for CVD treatment. (1) With the rapid advancement of science, there is a need to utilize network pharmacology approaches and multi-omics technologies, such as nutrigenomics, metabolomics, proteomics, gut microbial macrogenomics and immunomics, to reveal the physiological functions and mechanism explanations of TCM in combating CVD; (2) The metabolic, toxicity, and pharmacokinetic profiles of TCM fight against patients with CVD in clinical trials need to be further validated; (3) The construction of TCM resources for common quality standards to ensure active ingredient in TCM; (4) Research on active ingredients of TCM is limited by defects includes unstable chemical structure, low bioavailability and easy oxidation, and liposome embedding or nanoparticle formulation can be considered; (5) Development of CVD models with human disease characteristics for exploring the pharmacological activity of TCM, such as primate animal models that can avoid species barriers leading to ineffectiveness; (6) Designing TCM delivery systems to improve its stability, bioavailability, and efficacy in the gastrointestinal tract.

In conclusion, TCM possesses good anti-CVD effects and is an indispensable active drug for the treatment of CVD. Based on the latest evidence, this review summarized the pathogenesis of CVD and systematically analyzed and discussed the mechanisms of TCM in preventing CVD, as well as its clinical trials. This review aims to provide a scientific and effective comprehensive reference for TCM in CVD therapy and to better utilize and develop the treasures of TCM.
